# The Role of the Cytoskeleton in Regulating the Natural Killer Cell Immune Response in Health and Disease: From Signaling Dynamics to Function

**DOI:** 10.3389/fcell.2021.609532

**Published:** 2021-02-01

**Authors:** Aviad Ben-Shmuel, Batel Sabag, Guy Biber, Mira Barda-Saad

**Affiliations:** Laboratory of Molecular and Applied Immunology, The Mina and Everard Goodman Faculty of Life Sciences, Bar-Ilan University, Ramat Gan, Israel

**Keywords:** natural killer cells, actin, signaling, cytoskeleton, immune response

## Abstract

Natural killer (NK) cells are innate lymphoid cells, which play key roles in elimination of virally infected and malignant cells. The balance between activating and inhibitory signals derived from NK surface receptors govern the NK cell immune response. The cytoskeleton facilitates most NK cell effector functions, such as motility, infiltration, conjugation with target cells, immunological synapse assembly, and cytotoxicity. Though many studies have characterized signaling pathways that promote actin reorganization in immune cells, it is not completely clear how particular cytoskeletal architectures at the immunological synapse promote effector functions, and how cytoskeletal dynamics impact downstream signaling pathways and activation. Moreover, pioneering studies employing advanced imaging techniques have only begun to uncover the architectural complexity dictating the NK cell activation threshold; it is becoming clear that a distinct organization of the cytoskeleton and signaling receptors at the NK immunological synapse plays a decisive role in activation and tolerance. Here, we review the roles of the actin cytoskeleton in NK cells. We focus on how actin dynamics impact cytolytic granule secretion, NK cell motility, and NK cell infiltration through tissues into inflammatory sites. We will also describe the additional cytoskeletal components, non-muscle Myosin II and microtubules that play pivotal roles in NK cell activity. Furthermore, special emphasis will be placed on the role of the cytoskeleton in assembly of immunological synapses, and how mutations or downregulation of cytoskeletal accessory proteins impact NK cell function in health and disease.

## Introduction

Natural killer (NK) cells are innate lymphoid cells (ILCs) that constitute a major cellular component of the immune response. They play a pivotal role in eliminating cancerous and virally transformed cells, and may also participate in auto-immune diseases (Vivier et al., 2008). NK cells carry out their effector functions by directly killing target cells and by secreting modulatory cytokines. The cytotoxic pathway involves the release of lytic granules containing perforin and granzyme-B, or engagement of death receptors expressed on the surface of NK cells such as Fas ligand (FasL) and TNF-related apoptosis-inducing ligand (TRAIL) with cognate ligands expressed on target cells (Zamai et al., 1998; Dustin and Long, 2010). NK cells secrete IFN-γ, TNF-α, and GM-CSF to mediate their cytokine-based effector functions. Cytokines secreted by NK cells recruit and activate additional immune cells such as T-cells, B-cells, macrophages, and dendritic cells (Takeda, 1993; Martín-Fontecha et al., 2004; Walzer et al., 2005; Roetynck et al., 2006), and facilitate elimination of virally transformed target cells and cancer cells (Imai et al., 2000; Lee et al., 2007). In addition to their important roles in the innate immune response, NK cells have also been associated with adaptive immune responses, such as delivering more robust effector functions and proliferation in response to secondary Cytomegalovirus infection (Vivier et al., 2011).

The major mediator of NK cell effector activity is the cytoskeleton. Understanding the molecular regulation of the cytoskeleton in NK cells is critical, since NK cell effector functions are fundamentally linked with the cytoskeletal machinery. NK cells must circulate through blood and lymphatic vessels, traverse into tissues, recognize and eliminate relevant targets while sparing healthy cells, and recruit additional immune cells to relevant sites. Actin, which is the main component of the NK cytoskeleton, undergoes polymerization and depolymerization, from monomeric globular sub-units (G-actin) to ordered filaments (F-actin) and vice-versa during NK cell migration and conjugation with susceptible targets (Carpén et al., 1983). F-actin polymerization in NK cells is a dynamic event that is governed by activating or inhibitory signals delivered from cell surface receptors. Reorganization of the actin cytoskeleton is dependent on the activity of nucleating factors (NFs), which are responsible for direct actin nucleation. The central NFs include the Arp2/3 complex and formins. The activities of NFs are regulated by nucleation promoting factors (NPFs), such as members of the Wiskott–Aldrich Syndrome protein (WASp) family of proteins. Actin de-polymerizing factors, such as Coronin 1A, also play a direct regulatory role in NK cell cytotoxicity (Mace and Orange, 2014), as described below.

Another cytoskeletal component, non-muscle Myosin II (NM-II), the major isoform found in lymphocytes, regulates several functions of T-cells and NK cells. Myosin II utilizes ATP hydrolysis to generate contractile forces on actin filaments (Vicente-Manzanares et al., 2009). In T-cells, NM-II regulates motility (Jacobelli et al., 2004) and may regulate T-cell IS formation and stabilization (Kumari et al., 2012), however, this role remains uncertain (Jacobelli et al., 2004). While the role of Myosin in NK cell IS formation and stability remains an open question, research has revealed its importance in the cytotoxic activity of NK cells through forces exerted on lytic granules (Andzelm et al., 2007) and through regulation of cytoskeletal architecture to expedite degranulation (Carisey et al., 2018).

In addition to actin and Myosin, microtubule filaments play critical roles in NK cell and cytotoxic T cell (CTL) effector function. Microtubules are composed of alpha and beta tubulin heterodimers, and similarly to actin, undergo dynamic assembly and disassembly, which is regulated by a wide range of microtubule associated proteins (MAPS) (Akhmanova and Steinmetz, 2015). Microtubules facilitate the delivery of lytic granules to the synaptic cleft between NK cells and target cells, either directly through the centrosome or through microtubule associated motor proteins (Chen et al., 2006; Stinchcombe et al., 2006). Though studies explored microtubule-organizing center (MTOC) polarization in T-cells and possible roles the MTOC may play in maintaining IS stability (Kloc et al., 2014), it remains unclear if similar factors influence MTOC polarization in NK cells, and what other roles microtubule dynamics might serve at the NKIS aside from cargo delivery to the synaptic cleft.

It is well established that activating or inhibitory pathways differentially impact cytoskeletal rearrangement at the NKIS, yet the reciprocal role of cytoskeletal dynamics on NK signaling and maintenance of the activation threshold remains incompletely understood. Studies have, for example, suggested reciprocity in actin signaling in the context of integrin adhesion molecules. The integrin lymphocyte function-associated antigen 1 (LFA-1) induces “outside-in” signaling to promote actin polymerization during NK cell adhesion to target cells, and this actin polymerization subsequently increases LFA-1 mediated adhesion (Hoffmann et al., 2011). F-actin exerts physical forces on LFA-1 at the T-cell IS, ultimately influencing LFA-1 conformation during immunological synapse formation (Comrie et al., 2015a). F-actin dynamics may also exert forces on intracellular signaling molecules to impact NK cell output (Matalon et al., 2018). Thus, instead of merely acting as a static scaffold, the cytoskeleton may potentially possess a signaling role in NK cells via mechanotransduction. Moreover, an additional important question is how different cytoskeletal architecture at the NKIS influences signaling intensity and effector function. As we will discuss below, emerging super resolution imaging techniques are beginning to address this question, and demonstrate how distinct cytoskeletal arrangements influence receptor signaling and NK cell activation, with possible implications for NK cell priming and peripheral tolerance.

Due to the critical roles of the cytoskeleton in lymphocyte function, defects in cytoskeletal components may be detrimental to immune responses (described in detail below). Inhibition of actin polymerization has been shown to cause major defects in NK cell effector functions (Katz et al., 1982; Orange et al., 2002), and various immune deficiencies and diseases are attributed to defects of the cytoskeleton in immune cells (Matalon et al., 2013). Disorders affecting actin assembly in NK cells such as deficiencies in dedicator of cytokinesis 8 (DOCK-8), or in WASp, severely hamper NK cell responses (Orange et al., 2002; Mizesko et al., 2013). Myosin mutations have been shown to cause defects in NK cell activity in May-Hegglin anomaly patients (Sanborn et al., 2009). Furthermore, mutations that interfere with MTOC polarization to the IS also cause NK cell immunodeficiency in Hermansky–Pudlak syndrome subset 2 (HPS2) patients (Fontana et al., 2006).

In this review, we highlight the importance of the major cytoskeletal components for NK cell function. We emphasize how each cytoskeletal unit impacts different effector functions, and how together, they integrate to affect NK cell output. Furthermore, we address how cytoskeletal dynamics impact the architecture of the NKIS, and how they might also be involved in directly regulating signaling and tuning of the NK cell activation threshold. Finally, we discuss how dysregulation of the cytoskeleton results in primary immune deficiencies.

## Actin Cytoskeleton – a Key Signal Transducer and Regulator of NK Activation

### NK Signaling Cascades Leading to Cytoskeletal Recruitment and Reorganization

**FIGURE 1:**
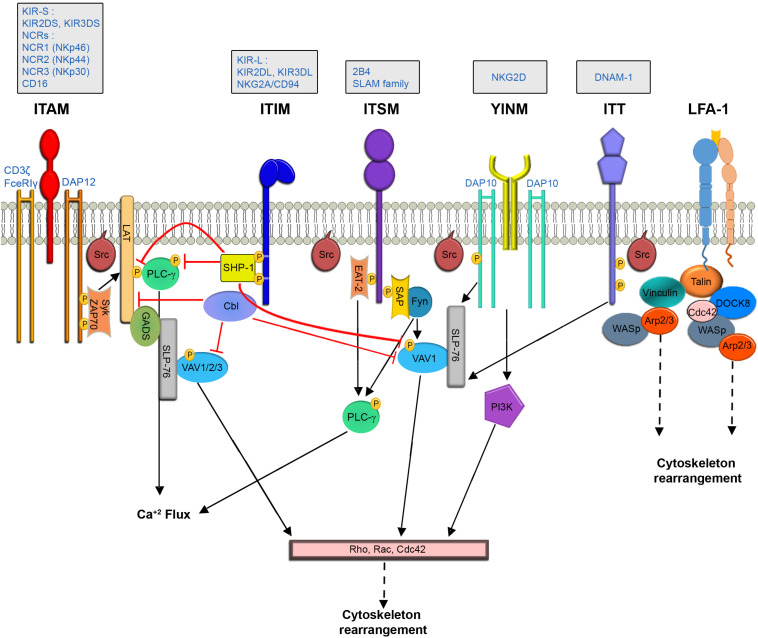
NK Cell signaling pathways leading to cytoskeletal rearrangement.

Natural killer cells express a large variety of germline-encoded receptors that regulate the immune response (Lanier, 1998). Importantly, cooperative signaling through ligation of activating receptor pairs (co-activation) appears necessary to fully stimulate NK cell activity (Bryceson et al., 2006; Kim et al., 2010). Signaling pathways in NK cells operate downstream of immunoreceptor tyrosine-based activation motifs (ITAMs) – and ITAM-independent motifs expressed on adaptor accessory molecules (Vély and Vivier, 2005). Ultimately both ITAM dependent and independent pathways initiate signaling cascades which affect actin polymerization and rearrangement, and converge into a cascade involving mitogen-activated protein kinases (MAPK), which are responsible for eliciting NK cell effector functions (Watzl and Long, 2010) (Figure 1).

#### The ITAM Pathway

The ITAM dependent pathway propagates downstream to activating receptors such as the activating killer-cell immunoglobulin-like receptor (KIR2DS), CD16, and the natural cytotoxicity receptors [NCRs- NCR1 (NKp46), NCR2 (NKp44) and NCR3 (NKp30) (Moretta et al., 2002)], which are associated with adaptor proteins such as CD3ζ, FceRIγ, and DAP12 (Lanier, 2003).

The SH2 domain containing leukocyte protein of the 76 kDa (SLP-76) adaptor protein promotes actin reorganization by facilitating interactions between VAV1, which is a guanine nucleotide exchange factor (GEF) for the Rho protein family, and the non-catalytic adaptor region of tyrosine kinase adaptor protein 1 (Nck) (Barda-saad et al., 2010; Pauker and Barda-Saad, 2011). Since VAV proteins serve as GEFs for the Rho GTPases Rac1 and Rho family GTPase Cdc42, which are critical for actin reorganization (Tapon, 1997), impairment of their activity has critical effects on NK effector function (Billadeau et al., 1998; Cella et al., 2004; Graham et al., 2006). During ITAM dependent signaling in NK cells, linker for activation of T cells (LAT) (Jevremovic et al., 1999; Matalon et al., 2016) and SLP-76 (Binstadt et al., 1998) couple upstream signaling events to downstream signaling proteins and complexes, which induce actin rearrangement. LAT was also shown to be a critical factor in NK cell activation by facilitating Phospholipase C-gamma (PLCγ) recruitment to the cell membrane (Jevremovic et al., 1999).

An additional signaling molecule that mediates actin reorganization at the NKIS is phosphatidylinositol 3-kinase (PI3K) (Cella et al., 2004). PI3K catalyzes production of phosphatidyl-inositol-3, 4,5-trisphosphate (PIP3), which is important for the recruitment of PH domain-containing proteins such as PLCγ and VAV1 (Han, 1998) to the immunological synapse. In NK cells, PI3K induces actin reorganization via STKs p21-activated kinase 1 (PAK1) (Papakonstanti and Stournaras, 2002).

#### ITAM Independent Pathways

In contrast to ITAM dependent cascades, NKG2D signaling is independent of LAT (Billadeau et al., 2003). The NKG2D receptor is associated with the DAP10 adaptor, which expresses a YINM motif (Upshaw et al., 2006). DAP10 can directly bind PI3K and the Grb2 adaptor. Grb2 recruits VAV1, initiating downstream actin re-organization. SLP-76 phosphorylation plays a crucial role in NK cell intracellular calcium level elevation, and activation in the context of NKG2D and 2B4 signaling. This activation causes a substantial increase in the phosphorylation of VAV1, leading to actin rearrangement (Kim and Long, 2012). Additional receptors that do not operate through ITAM motifs contain immunoreceptor tyrosine base switch motifs (ITSM) on their cytoplasmic tails, such as the receptor, 2B4 (Sidorenko and Clark, 2003). These signaling lymphocytic activation molecule (SLAM) family receptors can bind adaptors such as SLAM-associated protein (SAP) (Sayos et al., 1998) and Ewing’s sarcoma-associated transcript-2 (EAT-2) (Eissmann and Watzl, 2006) (in humans). SAP can activate NK cells by recruiting the Protein Tyrosine Kinase (PTK), Fyn (Latour et al., 2003). Fyn subsequently phosphorylates and activates VAV1, or phosphorylates and inactivates the SH2 domain-containing inositol 5′ phosphatase-1 (SHIP-1) (Eissmann et al., 2005; Dong et al., 2012). EAT and SAP provide synergistic effects for 2B4 activation, as double SAP/EAT-2 deficient mice displayed greater 2B4 mediated inhibition than those deficient in one of the adaptors alone (Dong et al., 2012); these activities include phosphorylation and activation of LAT, VAV1, PLCγ1, and Grb-2 (Watzl et al., 2000; Chen et al., 2004), which are involved in actin polymerization and rearrangement.

#### Integrin Signaling

LFA-1 engagement with its cognate ligand, intercellular adhesion molecule 1 (ICAM-1) results in tyrosine phosphorylation of VAV1, which increases when both LFA-1 and the 2B4 are engaged with their ligands (Riteau et al., 2003). Additional cascades downstream to LFA-1 engagement in NK cells include tyrosine phosphorylation of T cell antigen receptor (TCR) ζ-chain, Syk, paxillin, and PLC-γ1/2 (March and Long, 2011), leading to the actin dependent process of granule polarization to the NKIS. Paxillin phosphorylation by proline-rich tyrosine kinase-2 (Pyk2) in NK cells was also shown downstream to β1 integrins (Gismondi et al., 1997). Furthermore, following engagement of LFA-1 with ICAM-1, the focal adhesion (FA) protein talin localizes to sites of LFA-1 engagement. Talin recruits Arp2/3, and binds phosphatidylinositol 4-phosphate 5-kinase (PIPKI). This leads to a local increase in phosphatidylinositol-4, 5-bisphosphate PIP(2), recruiting WASp, which facilitates actin polymerization through Arp2/3 (Mace et al., 2010). Zhang et al. (2015) additionally elucidated the signaling pathways leading to actin rearrangement downstream to the DNAX accessory molecule-1 (DNAM-1) receptor, which contains a tyrosine and asparagine based ITT-like motif. In this pathway, triggering of DNAM-1 leads to phosphorylation of the ITT motif by a Src family kinase, recruitment of the adaptor Grb2, and activation of VAV1 and PLCγ-1, leading to actin rearrangement (Zhang et al., 2015).

### WASp and WIP Mediated Regulation of NK Cell Cytoskeletal Dynamics and Function

Nucleating factors, such as Arp2/3 and formins, directly affect actin polymerization, and nucleation-promoting factors such as WASp and the WASp-family verprolin-homologous protein (WAVE) bind and regulate actin nucleating factors through verprolin, central, acidic (VCA) domains (Chereau et al., 2005). The central NPF families include WASp, WAVE, SCAR homolog (WASH), the WASp homolog associated with actin, membranes and microtubules (WHAMM), and the junction-mediating and -regulatory protein (JMY). The Arp2/3 complex promotes cross-linking of actin filaments, and thereby promotes formation of an actin meshwork at the leading edge of cells (Mullins et al., 1998). This activity by Arp2/3 drives cell motility and spreading at the NKIS (Butler and Cooper, 2009). Recent super resolution microscopy experiments (described in the following sections) also revealed that Arp2/3 branching activity at localized actin structures (actin puncta) at the NKIS mediated actin remodeling, which facilitates cytotoxicity (Carisey et al., 2018). Formins aid in the creation of a subset actin filaments that are not barbed and have specific functions- such as the creation of stress fibers and endosome trafficking, as well as formation of filopodia and micro-spikes at the edge of the expanding cell membrane (Gasman et al., 2003; Wallar and Alberts, 2003). Formins play various roles in T-cell synapse architecture and dynamics (Eisenmann et al., 2007; Gomez et al., 2007; Murugesan et al., 2016), however in NK cells, the function of formins has not been as extensively explored. It does appear, however, that formin family members such as hDia1 facilitate NK cell adhesion, chemotaxis, and chemokine-induced signaling (Butler and Cooper, 2009), in addition to promoting microtubule dependent movement and polarization of cytolytic granules (Butler and Cooper, 2009).

#### WASp

WASp contains several domains that dictate its function and regulation: WASp homolog (WH1) domain, a basic region (B), GTPase binding (GBD) domain, poly proline region, and a VCA domain (Thrasher and Burns, 2010). Under basal conditions, the WASp VCA domain lies in close proximity to the GBD domain, inhibiting binding of Arp 2/3 (Kim et al., 2000). Binding of GTP-Cdc42 to the WASp GBD domain releases WASp from auto inhibition, and enables binding of Arp2/3 to the VCA domain and initiation of actin nucleation (Abdul-Manan et al., 1999). Phosphorylation of tyrosine 291 (Tyr 291) in the GBD domain was also shown to augment WASp activity (Cory et al., 2002).

Upon NK cell activation, WASp forms a multi-protein complex with WASp-interacting protein (WIP), actin, and Myosin, and these associations are abrogated during NK cell inhibition (Krzewski et al., 2006). Moreover, NK cell stimulation (either through CD16 or through chemokine receptors and β_1_ and β_2_ integrin families) leads to WASp phosphorylation, strongly suggesting that these mechanisms are required for WASp dependent NK cell cytotoxicity (Orange et al., 2003; Gismondi et al., 2004; Andzelm et al., 2007; Stabile et al., 2010). In murine NK cells, engagement of LFA-1 with ICAM-1 results in WASp recruitment to the site of contact, activation of Arp2/3, and actin polymerization at the LFA-1 contact site (Mace et al., 2010).

WASp impacts multiple facets of NK cell activity which depend on cytoskeletal turnover, such as migration, IS formation, and cytotoxicity. An absence of WASp in NK cells disrupts formation of the typical cytotoxic NKIS due to a significant decrease in actin accumulation (Orange et al., 2002), and also leads to a reduction in lytic granule polarization and NK cytotoxicity (Orange et al., 2002; Huang et al., 2005). NK cells also require the function of integrins and other adhesion molecules to create conjugates with target cells and stabilize the NKIS (Davis, 2009). Studies employing NK cells with WASp mutations that lead to a low yet detectable level of WASp, and WASp mutations that completely abrogate WASp expression demonstrate a decrease in the ability of NK cells to form conjugates and hence to initiate targeted cytotoxicity; these findings suggest a possible regulatory role for WASp in cytoskeleton organization which may affect adhesion molecules on the NK cell membrane (Gismondi et al., 2004). A reciprocal regulatory mechanism may exist between WASp and actin turnover, because treatment of NK cells with cytochalasin D, an actin polymerization inhibitor, results in decreased WASp, F-actin, and perforin accumulation at the activating NKIS (Boztug et al., 2008). These results suggest a positive feedback mechanism in which WASp-dependent actin polymerization is responsible for further accumulation of WASp at the NKIS. In T cells, WASp plays a role in formation of dense actin centers, or “actin foci,” which enhance downstream signaling. It would be informative to study whether WASp functions similarly in NK cells to promote formation of actin foci at the NKIS, as dense actin puncta are observed during NK cell activation and degranulation (Carisey et al., 2018), and nanoscale organization of NK cell receptors are also dependent on local cytoskeletal dynamics (Pageon et al., 2013b).

Impaired WASp activity also negatively impacts NK cell motility. NK cells from WAS and XLT patients demonstrate impaired ICAM-1, VCAM-1, and endothelial cell mediated migration (Stabile et al., 2010). The defective chemokine induced migration of these cells is correlated with reduced expression of the activated form of the β_2_ integrin subunit, and the decreased adhesion to ICAM-1 and VCAM-1. Thus WASp signaling pathways are essential for NK cell LFA-1-mediated migration in response to chemokine receptor-induced inside-out signaling (Stabile et al., 2010).

Defects in cytotoxicity, cytokine secretion, and migration in WASp knockout NK cells may also be due in part to upregulation of NK cell checkpoint markers, which might down modulate the NK cell response, such as LAG-3 and KLRG1 (Kritikou et al., 2016). It appears, however, that IL-2 uptake by NK cells bypasses defects in WASp expression (Gismondi et al., 2004), suggesting alternative mechanisms which compensate for WASp function, probably through WAVE-2 actin reorganization (Orange et al., 2011). Nonetheless, WASp function is critical for NK cell effector activity, and its loss in NK cells was also recently shown to promote tumor growth *in vivo* (Catucci et al., 2014).

#### WIP

WIP functions as a WASp stabilizing protein, and prevents WASp degradation in immune cells (de la Fuente et al., 2007; Noy et al., 2012; Pauker et al., 2012; Reicher et al., 2012; Fried et al., 2014b). Mutations in the WASp WH1 domain, which mediates its interaction with WIP, are associated with several phenotypes in WAS patients (Imai et al., 2003). As mentioned above, NK cell activation induces formation of a multi protein complex consisting of WASp, WIP, actin, and Myosin (Krzewski et al., 2006) which facilitates actin reorganization and NK cell effector function. WIP is crucial for formation of this complex, as it recruits NM-IIA and actin to the complex, and disruption of its expression abrogates complex formation. WIP also has its own distinct role in NK cell cytotoxicity; WIP knockdown results in a significant reduction of cytotoxicity, while WIP overexpression enhances NK cell activity (Krzewski et al., 2006). The role of WIP in NK cell cytotoxicity is suggested to result from WIP colocalization with lytic granules in both resting and activated NK cells, a process that was shown to be independent of WASp (Krzewski et al., 2008; Fried et al., 2014a). WIP knockdown inhibits the observed granule polarization upon NK cell activation, suggesting that co-localized WIP and lytic granules are polarized to the NKIS in a WIP-dependent fashion. In contrast to WASp deficiency, knockdown of WIP does not disrupt NK cell conjugation to their targets, thereby indicating that WASp and WIP have distinct functions in the control of NK cell cytotoxicity.

### Additional Factors Mediate Cytoskeletal Reorganization at the NKIS

Other cytoskeletal regulators have been described in the context of NK cell activity, albeit not extensively. WAVE is a WASp family protein that also regulates cytoskeletal re-arrangement (Miki et al., 1998). The WAVE2 isoform is the most abundant isoform in hematopoietic cells (Suetsugu et al., 1999). The VCA region of WAVE2 is implicated in binding Arp2/3 and actin monomers, subsequently leading to induction of actin polymerization (Takenawa and Suetsugu, 2007). Experiments in T-cells demonstrated an important role for WAVE2 in actin re-organization and adhesion; WAVE2 was shown to migrate to the IS, and WAVE2 gene silencing leads to a decrease in actin polymerization, decreased lamellopodia formation during T-cell spreading, and reduction in the ability of T-cells to form conjugates with targets (Nolz et al., 2006, 2007, 2008; Sims et al., 2007; Reicher et al., 2012; Pauker et al., 2014). In NK cells, WAVE2 activity has not been extensively studied. WAVE2 can compensate for WASp deficiency, as IL-2 administration bypasses WASp inactivity (either in WAS patients or in WASp deficient and inhibited NK cells) by activating WAVE2, thereby restoring actin polymerization at the NK cell IS and restoring NK cell cytotoxic activity (Orange et al., 2011). This suggests a bypass mechanism(s) in NK cells, operating through IL-2 to ensure actin assembly.

The DOCK GEFs, DOCK2, DOCK8, and RAS guanyl-releasing protein 1 (RASGRP1), are also cytoskeletal regulating proteins, which were shown to play roles in NK cell actin rearrangement. DOCK2 functions as a GEF for the Rho family protein Rac (Brugnera et al., 2002). DOCK2 deficient NK cells lose cytotoxic capacity against target cells due to impaired actin polymerization and subsequent lytic synapse formation (Sakai et al., 2013). DOCK8 functions as a GEF for Rac and CDC42 (Harada et al., 2012). In NK cells, DOCK8 interacts with talin and WASp, and DOCK8 deficiency also results in impaired NK cell cytolytic function and adhesion due to impaired F-actin accumulation at the NKIS (Ham et al., 2013; Mizesko et al., 2013). RASGRP1 serves as a GEF for Ras GTPase, thereby promoting lymphocyte activation and differentiation (Roose and Weiss, 2000; Stone et al., 2000). Recent studies in a young patient with RASGRP1 deficiency demonstrated impaired immune cell functions in T, B, and NK cells (Salzer et al., 2016). The patient’s NK cells produced normal amounts of the effector granzyme B and perforin proteins, but demonstrated impaired cytotoxic ability due to defective IS formation. This defective IS was characterized by poor F-actin accumulation, MTOC polarization, and recruitment of lytic granules at the MTOC.

Additional cytoskeletal regulatory proteins such as WASH, hematopoietic lineage cell-specific protein 1 (HS1), and IQ domain-containing GTPase-activating protein 1 (IQGAP1) were identified to play roles in NK cell effector activity by regulating lytic granule dynamics, IS assembly, trans endothelial migration (Butler et al., 2008; Kanwar and Wilkins, 2011; Mukherjee et al., 2015; Huang et al., 2016; Abel et al., 2018). Further study is required to better understand how these proteins operate in the context of NK cell effector activity.

#### Coronin 1A

In addition to factors promoting actin polymerization, actin de-polymerizing proteins are also critical for actin rearrangement, as actin assembly and disassembly are the two opposing processes that drive actin dynamics at the NKIS. Moreover, since lytic granules must traverse a dense actin network at the NKIS in order to reach their destination, a regulated mechanism must exist to promote localized actin disassembly. As mentioned above, Coronin 1A, a hematopoietic regulator of actin, which promotes actin disassembly (Kueh et al., 2008), plays a critical role in NK cell cytotoxicity. Coronin 1A associates with Arp2/3 and inhibits its function, while stimulating Cofilin activity, thereby promoting actin filament de-polymerization (Humphries et al., 2002; Kueh et al., 2008). Specifically, Coronin 1A was shown to localize at the NKIS and reconstruct the actin meshwork to permit lytic granule release (Mace and Orange, 2014). Cells lacking Coronin 1A display impaired lytic granule release and thus cytotoxic deficiencies due to their inability to induce target cell death (Mace and Orange, 2014). The presence of an actin de-polymerizing factor such as Coronin 1A at the activating NKIS, where significant actin recruitment and assembly takes place, highlights the complex and dynamic nature of actin regulation that ensures NK cell effector functions. This mechanism may also safeguard against potential bystander cell cytotoxicity by limiting the space of lytic granule delivery at the synaptic cleft. Coronin 1A and other actin de-polymerizing factors may also play an opposing role at inhibitory NK cell synapses where actin assembly and dynamics differ greatly. It is possible that the mode of regulation at the inhibitory NKIS involves not only simply blocking activating signals of actin nucleation, but also deconstructing the existing actin architecture in order to ensure target cell survival.

## The Actin Cytoskeleton and NK Cell Function

### NK Cell Motility and Infiltration

Natural killer cells must retain high motility to navigate through the circulatory system and tissues and reach areas of infection (Timonen, 1997). NK cells are exceedingly motile, an important characteristic that facilitates movement through lymphoid organs and their ability to patrol peripheral tissues and organs for immuno-surveillance (Garrod et al., 2007). Migrating leukocytes change morphologically during migration as a result of actin dynamics as well as contraction of acto-Myosin-associated arcs [curved bundles of actin filaments with a periodicity of Myosin and alpha-actinin (Tojkander et al., 2012)], creating a leading edge rich in F-actin known as the lamellipodium, and a trailing edge poor in F-actin and rich in adhesion molecules known as the uropod (Vicente-Manzanares and Sánchez-Madrid, 2004). Several studies demonstrate the impaired motility of NK cells when cytoskeletal integrity is compromised. This is especially evident in NK cells deficient in WASp and other critical cytoskeletal regulators such as RASGRP1 and DOCK2 as will be described in more detail in the next sections. Artificial down modulation of actin dynamics through inhibition of Arp2/3 or hDia1, abrogates NK cell chemotaxis (Butler and Cooper, 2009) and recent studies examining pathological conditions such as aging and cancer additionally show that NK cells from aged mice contain lower levels of β-actin, reducing their migration to draining lymph nodes during viral infection (Duan et al., 2017). In a further example, down modulation of F-actin polarization in NK cells by colon tumors serves to inhibit NK cell migration (Wang et al., 2016). NK cells must also attach to blood vessels and cross endothelial barriers to reach target tissues. Attachment of the NK cells to endothelial cells occurs through NK cell adhesion molecules such as the integrins LFA-1 and very late antigen-4 (VLA-4), which bind to endothelial markers such as ICAM-1 and VCAM-1, respectively (Allavena, 1991; Fogler et al., 1996). To effectively infiltrate tissues, cellular actin reorganization must occur to generate proper forces to “squeeze” the cell through the narrow spaces of the endothelium (Worthylake and Burridge, 2001; Lämmermann et al., 2008). Blocking F-actin reorganization induced by the chemokines CX3CL1 and CCL26 prevents NK cells from undergoing the morphological changes required for proper tissue extravasation (El-Shazly et al., 2013). Accordingly, HS1 deficiency in NK cells also down regulates their capacity for trans-endothelial migration (Mukherjee et al., 2015).

Few studies examined the role of Myosin motor function in NK motility. It is possible that the balance between activating and inhibitory signaling in NK cells regulates Myosin activity to promote a stop signal, i.e., inducing NKIS formation instead of NK cell migration, as is the case for F-actin (Culley et al., 2009). Further studies could show the distribution of Myosin in NK cells and whether, in analogy to migrating T-cells, it is situated in the uropod of motile cells, and could elucidate how NK cell activation and IS formation influence Myosin placement and activity.

### Organization of Signaling Receptors at the Activating NKIS

**FIGURE 2:**
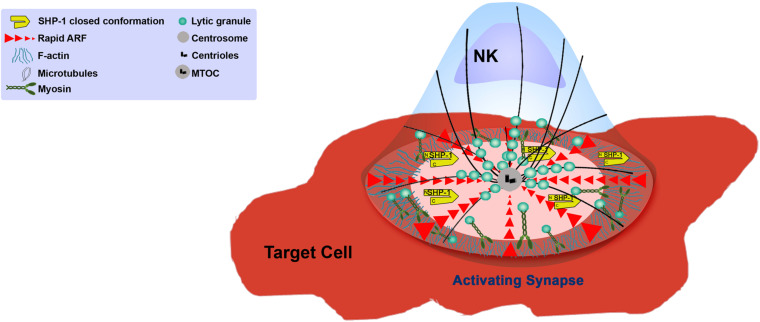
Function of the NK cell cytoskeleton during activation.

**FIGURE 3:**
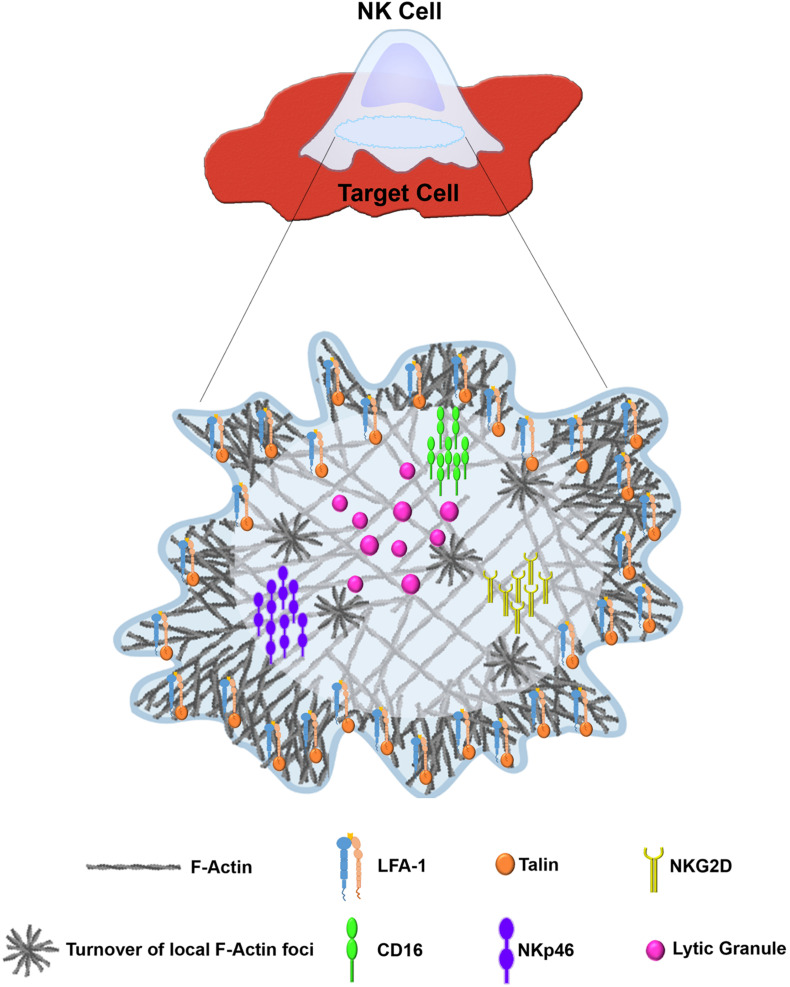
Distinct architecture of the lytic NKIS. Figure was generated using BioRender (https://biorender.com).

The creation of the NKIS requires intimate contact between the NK cell and its target. Large scale rearrangement of the actin cytoskeleton at the NKIS serves multiple purposes (Vyas et al., 2001) (Figure 2), namely (a) adhesion between the NK cell and the target cell to ensure the longevity and stability of the contact, (b) assembly of signaling complexes, and (c) controlled killing of the target cell (Vyas et al., 2002b). The NKIS shares some characteristics with the T-cell IS, though it is also characterized by its own distinct features, and performs several functions to properly integrate signals that identify transformed or virally infected cells (Figure 3). These include receptor-ligand recognition, creation of signaling clusters for signal enhancement, co-stimulation by co-stimulatory ligands, directed cytotoxicity, cell to cell protein transfer, signal termination, and in the case of inhibitory synapses that promote tolerance, inhibition of activation (Orange, 2008).

The T-cell IS contains areas with distinct protein compositions and actin dynamics, termed supramolecular activation clusters (SMACs). The SMACs roughly correspond to areas of distinct actin reorganization that are observed in migrating cells; the outermost peripheral “ring” known as the distal SMAC (dSMAC) and the more inner ring called the peripheral SMAC (pSMAC) may be analogous to the lamellipodium and the lamellum, respectively (Dustin et al., 2010). Thus, the dSMAC is rich in Arp2/3 and cofilin, leading to cycles of protrusion and retraction (Sims et al., 2007), and tropoMyosin localizes in the pSMAC where actoMyosin networks provide contractile forces and adhesion molecules mediate attachment to the substrate (Ponti et al., 2004; Sims et al., 2007). The central SMAC (cSMAC) can be divided into two areas: the endo-cSMAC where TCR and CD28 signaling persist, and the exo-cSMAC, which is an actin-depleted zone containing TCR-rich extracellular vesicles that bud from the plasma membrane, and where the signaling region terminates (Choudhuri et al., 2014; Dustin, 2014). Initially, the immature activating T-cell IS contains vital signaling molecules such as the TCR in the peripheral SMAC (pSMAC) and adhesion molecules in the cSMAC of the synapse; during IS maturation, the dominant signaling molecules (i.e., TCR–MHC peptide interactions) migrate toward the center of the synapse, and the adhesion molecules (LFA-1-ICAM-1 interactions) localize in the pSMAC, while the CD45 membrane tyrosine phosphatase localizes in the distal SMAC (dSMAC) (Monks et al., 1998; Johnson et al., 2000). Ligation of TCR with MHC:peptide complexes induces formation of TCR micro-clusters which ultimately initiate a protein tyrosine kinase cascade resulting in T-cell activation (Dustin, 2014). Proper assembly of the IS, and subsequent signaling cascade initiation are thus highly dependent on actin reorganization (Barda-Saad et al., 2005; Yi et al., 2012; Hammer et al., 2019). The T-cell IS is characterized by rapid actin turnover at the dSMAC driven by WAVE2 and Arp2/3 activity, arcs of contracting actin filaments and Myosin at the pSMAC, which are generated by formin activity at the outer edge of the IS, and an actin poor cSMAC (Murugesan et al., 2016; Hammer et al., 2019). Additional structures at the T-cell IS include actin foci at the dSMAC and pSMAC generated via WASp and Arp2/3, which were shown to activate T-cells through the PLCγ pathway (Kumari et al., 2015).

The activating NKIS involves the accumulation of F-actin atthe cell–cell junction, eliciting morphological changes in the NK cell and creating a radially symmetric and stable contact site (Orange et al., 2002; Wulfing et al., 2003; Culley et al., 2009) that is composed of the pSMAC and the cSMAC. Collectively, the formation of the activating NKIS consists of two major steps: (1) rapid accumulation of F-actin and integrins in the pSMAC, and (2) slow polarization of the cytolytic proteins, e.g., perforin and other key signaling molecules (Davis et al., 1999). The NK cell activating synapse initiates formation of a dense ring of actin, LFA-1, and talin-1 around the cSMAC (Vyas et al., 2001). This primary actin-induced spreading response is very sensitive to the balance between activating and inhibitory ligands; inhibitory ligands were shown to inhibit the spreading response even if it was already initiated under activating conditions (Culley et al., 2009; Abeyweera et al., 2011). Accumulation of signaling molecules at the NKIS was shown to enhance NK signaling (Varma et al., 2006; Giurisato et al., 2007). F-actin polymerization is thought to play an important role in this signaling cluster assembly. In particular, LFA-1, MAC-1 and CD2 function as adhesion molecules in NK cells, and were shown to depend on actin polymerization for polarization and clustering at the IS (Orange et al., 2003). In addition, the 2B4 receptor is expressed on NK cells and plays a role in generating cytotoxicity and cytokine production (Nakajima et al., 1999), and it was shown that its recruitment and phosphorylation are dependent on actin dynamics (Watzl and Long, 2003). Accordingly, considering the vast and versatile function of actin in the formation and function of the IS, accumulation of F-actin at the IS decreases in the presence of actin inhibitors or in the absence of crucial actin regulators such as WASp, leading to a decrease in adhesion necessary for conjugate formation and cytotoxicity (Orange et al., 2002; Wulfing et al., 2003).

The mechanisms by which NKIS architecture influences NK cell signaling (both activation and inhibition) are complex given the large array of activating and inhibitory receptors and co-receptors. Elucidation of the organization of signaling molecules at the NKIS was facilitated through utilization of advanced and super resolution microscopy experiments. Oszmiana et al. (2016) demonstrated that the activating receptor KIR2DS1 and the DAP12 signaling adaptor associate during receptor ligation, generating large receptor clusters. These large clusters favor phosphorylation of ZAP-70 and NK cell activation (Oszmiana et al., 2016). Thus, it appears that the size of signaling clusters affects signal strength and sways NK cells toward either activation or inhibition. It is possible that these mechanisms occur to overcome large intervals between ligands on target cells, because activation of NK cells decreases with increased spacing of ligands for CD16 (Delcassian et al., 2013). NKG2D was also previously shown to organize into microclusters at the activating NKIS, and this organization depends on actin remodeling (Abeyweera et al., 2011). Furthermore, recent studies also elucidated the organization of the NKG2D receptor on the surface of NK cells following stimulation of its ligands, MHC class I polypeptide-related sequence A (MICA) or UL16 binding protein 1 (ULBP1) (Bálint et al., 2018). ULBP1, and not MICA, induces large complexes of NKG2D and the IL-2/15 receptor subunits, demonstrating the ability of different ligands to differentially activate NK cells. The different organization of NKG2D in response to its ligands could potentially be due to its different affinities for ULBP1 and MICA, however, this remains unclear. In addition, NKp46 appears to cluster during NK cell stimulation (Hadad et al., 2015), and CD16 also forms clusters upon NK cell stimulation that are eliminated during inhibition of actin cytoskeletal reorganization (Liu et al., 2012). It is still incompletely understood how distinct cytoskeletal structures mediate the different organizations of NK cell receptors in response to different ligands. As discussed in the following sections, multiple studies implemented advanced microscopic techniques that delineated novel cytoskeletal structures at the NKIS; these structures proved indispensable for proper NK cell activity. Therefore, the arrangement of receptors may be linked to the observed cytoskeletal organization at different synapses. It is possible that distinct actin architectures at the NKIS enhance or reduce signaling propagation by influencing organization of particular receptors.

### Cytotoxicity

#### Myosin

NM-II is a motor protein, which belongs to a class of molecular motor proteins that transduce cellular free-energy into motion. There are three members of non-muscle Myosin II family: Myosin IIA, Myosin IIB, and Myosin IIC (Maravillas-Montero and Santos-Argumedo, 2012).

The dominant Myosin isoform present in hematopoietic cells is non-muscle Myosin IIA (NM-IIA) (Maravillas-Montero and Santos-Argumedo, 2012). NM-IIA is a hexamer that contains two heavy chains with globular “heads” in the N terminus that bind actin filaments and mediate ATPase activity, which drives contractile forces along actin filaments. The two regulatory light chains (RLC) and two essential lights chains (ELC) regulate Myosin function and structural stability, respectively (Vicente-Manzanares et al., 2009).

Several studies examined the activation induced role and regulation of Myosin at the NKIS. As mentioned above, activation of NK cells induces formation of a multiprotein complex comprised of Myosin with WIP, WASp, and actin (Krzewski et al., 2006). In the same study, inhibitory signals abrogated the recruitment of Myosin and actin to WIP/WASp. Myosin recruitment to this complex, and subsequent recruitment to the NKIS, was shown to depend on WIP. When this multiprotein complex was disrupted, NK cytotoxic potential was greatly decreased. It was suggested that Myosin motor function may aid in recruiting WASp and WIP to the NKIS, where further actin polymerization and branching occur. It is also possible that Myosin can be recruited through the WIP/WASp complex by actin to the NKIS, and associates at the interface with lytic granules for directed granule secretion. This would also explain the loss of cytotoxicity upon abrogation of the complex.

Andzelm et al. (2007) demonstrated that while Myosin is crucial for the exocytosis of lytic granules at the NKIS, it is dispensable for NK/target cell conjugation and NKIS formation. In terms of NKIS maturation in this study, only CD2, perforin, and actin accumulation were assessed. It would be interesting to examine, in a similar fashion, if important downstream signaling molecules crucial for activation are impacted as a result of NM-IIA inhibition. It is possible that though conjugation is seemingly unaffected, reduction in NK cell cytotoxicity is also a result of impaired signaling resulting from NM-IIA ATPase activity. Further studies evaluating the role of NM-IIA dynamics on NK cell signaling will need to be conducted to answer these questions. The mechanism by which NM-IIA facilitates NK cell cytotoxicity was subsequently shown by Sanborn et al. (2009), who demonstrated that NM-IIA physically associates with lytic granules and augments granule association with actin filaments at the IS; this process ultimately expedites granule release at the synaptic cleft. Mechanistically, the Myosin IIA tailpiece is constitutively phosphorylated in NK cells on Serine 1943 (S1943); this phosphorylation is critical for Myosin association with lytic granules and NK cell cytotoxicity (Sanborn et al., 2011). The kinase that phosphorylates S1943 may be casein kinase II (Dulyaninova et al., 2005), though this is yet to be resolved in NK cells (Sanborn et al., 2011). In addition to these findings, NM-IIA was shown to recruit Ras-related protein Rab-27A and Protein unc-13 homolog D (munc13-4) to lytic granules upon NK cell stimulation (Wood et al., 2009). Rab-27A regulates vesicle trafficking, and munc13-4 regulates fusion of granules with the plasma membrane (Ménasché et al., 2000; Feldmann et al., 2003). Inhibition of NM-IIA abrogates Rab-27A and munc13-4 recruitment to lytic vesicles, down-modulating NK cell cytotoxicity (Wood et al., 2009). In agreement with the importance of NM-IIA in granule exocytosis, silencing of its co-chaperone UNC-45A in NK cells severely impairs degranulation by impacting acto-Myosin contraction (Iizuka et al., 2015). Moreover, in NK cells, contractile forces exerted by NM-IIA are critical for local nano-scale actin dynamics at the NKIS. These local events of actin reorganization define the overall synaptic architecture that is critical for NK cell cytotoxicity (Carisey et al., 2018). Nevertheless, the molecular steps that precede NM-IIA association to lytic granules and their directed delivery through the synaptic cleft are not completely understood. The overall signaling regulation of these processes have yet to be understood in context of both NK cell activation and inhibition.

NM-IIA was shown to play a critical role in T-cell motility. NM-IIA heavy chain (NMMHC-IIA) localizes in the Uropod of motile T-cells and is recruited to the interface of T-cell/APC synapses (Jacobelli et al., 2004). Inhibition of NM-IIA with blebbistatin arrests T-cell polarity and migration, and induces cell rounding. Additionally in T-cells, engagement of the TCR induces phosphorylation of NMMHC-IIA on threonine 1939, which reduces NM-IIA contractile activity, thereby potentially inducing a T-cell stop signal for locomotion (Jacobelli et al., 2004). Thus, NM-IIA may play an important role in inducing signals to transition lymphocytes from synapse formation to movement. This role of NM-IIA is perhaps mediated through LFA-1, as interactions between NMMHC-IIA and LFA-1 were shown to facilitate LFA-1 dissociation during T-cell migration (Morin et al., 2008). It would be interesting to examine whether and how NM-IIA performs similar roles in NK cells, which depend on a multitude of signaling inputs from various surface receptors. It may be possible that localized co-activating signals in NK cells are required to regulate Myosin activity in order to promote NK cell synapse formation, however, additional work is required to elucidate these mechanisms.

#### Microtubules

The microtubule cytoskeleton is another significant component of NK cell function. Microtubule filaments are assembled via heterodimers of αβ tubulin. Microtubule polymerization is driven by hydrolysis of GTP bound to the αβ tubulin dimer (Akhmanova and Steinmetz, 2015). The origin of microtubule polymerization is the MTOC, which consists of the centrosome and pericentriolar material (PCM) (Kloc et al., 2014). Hence, microtubule filaments can extend from the MTOC and disassemble in response to stimuli and regulatory proteins. Microtubule plus-end-tracking proteins (TIPs) can associate with growing microtubule ends and increase the polymerization rate (Schuyler and Pellman, 2001). These include microtubule polymerases such as the XMAP215 family and microtubule end binding proteins (EBs) such as EB1 (Zanic et al., 2013), as well as cytoplasmic linker protein (CLIP)-associated proteins (CLASPs) (Galjart, 2005). Furthermore, molecular motor proteins such as Dynein can associate with microtubules and stabilize them (Hendricks et al., 2012). Other proteins destabilize microtubules and enhance microtubule depolymerization by removing terminal tubulin caps. These include, for example, the microtubule depolymerases such as those of the Kinesin family (Kinesin-13, 8, and 14) (Desai et al., 1999; Sproul et al., 2005; Gardner et al., 2011).

In the context of immune cell function, there has been great interest in understanding the molecular mechanisms governing microtubule, and specifically MTOC, orientation toward the IS during T-cell/NK cell interactions with targets, and the possible function of the MTOC in IS stability. Furthermore, the process of lytic granule convergence onto the MTOC, which ensures directed cytotoxicity while preventing bystander cell killing is an ongoing field of investigation. Microtubules were shown to possess several diverse functions in T-cells. The most well-studied function of MTOC polarization in cytotoxic T-cells (CTLs) and NK cells is release of cytotoxic granules (Stinchcombe et al., 2006; Topham and Hewitt, 2009). Inhibition of MTOC polarization in NK cells disrupts cytotoxic capacity (Chen et al., 2007). Prior to MTOC polarization to the NKIS, lytic granules converge onto the MTOC via activity of Dynein motor proteins (Mentlik et al., 2010). One of the proteins that facilitates lytic granule convergence through Dynein in NK cells is the Hook-related protein 3 (HkRP3), which binds Dynein and mediates association between DOCK8 and the microtubule network (Ham et al., 2015). In addition, the small GTP binding protein, ADP-ribosylation factor-like 8b (Arl8b), binds Kinesin family member 5B (KIF5B), SifA, and Kinesin-interacting protein (SKIP), facilitating movement of the MTOC to the NKIS (Tuli et al., 2013). Another recently identified Kinesin motor protein that is important for NK cell cytotoxicity toward fungal pathogens is Eg5-Kinesin, which was shown to facilitate Dynein-mediated lytic granule convergence to the MTOC (Ogbomo et al., 2018). Recently, vasodilator-stimulated phosphoprotein (VASP), which is an actin regulatory protein belonging to the Ena/VASP family, was shown to play an important role in lytic granule convergence to the MTOC through actin filament assembly (Wilton and Billadeau, 2018). This mechanism of lytic granule convergence was recently shown to be of critical importance in NK cell biology, ensuring targeted cell lysis and preventing bystander cell death (Hsu et al., 2016). An additional member of the Ena/VASP actin regulators, EVL, was also recently shown to be recruited to the cytotoxic NKIS, where it is involved in maintaining adhesion between NK cells and targets, and in facilitating NK cell synapse maturation (Wilton et al., 2019). Lack of EVL in NK cells resulted in decreased actin generation at the NKIS and reduced NK cell killing. EVL operates downstream to the NKG2D-Grb2-VAV1 axis, where it recruits WASp and VASP to induce F-actin accumulation at the NKIS and facilitate effector functions (Wilton et al., 2019).

Different signaling pathways lead to lytic granule convergence and microtubule reorientation to the NKIS, but not to degranulation. For example, signaling from integrin molecules, such as β2 integrins, is sufficient to promote granule polarization to the NKIS (Barber et al., 2004). Zhang et al. (2014) additionally defined this signaling pathway by decoupling additional receptors, and demonstrated that it involves activation of integrin linked kinase (ILK), Pyk2, paxillin, Rho guanine nucleotide exchange factor 7 (RhoGEF7), Cdc42, and Par6. These results extend earlier descriptions of Pyk2 in the NK cytolytic response (Sancho et al., 2000). Additional defined signaling pathways required for polarization of the MTOC were described downstream to the CD28 receptor, and include activation of PI3K which leads to phosphorylation of extracellular signal-regulated kinase 2 (ERK2) (Chen et al., 2006). It should be noted, that this CD28 dependent signaling cascade (CD28-PI3K-ERK2) was described in the YTS cell line, and not other NK cell lines or primary cells. In addition to CD28, crosslinking of the activating NK cell receptors NKG2D, NKp30, NKp46, NKG2C/CD94, or 2B4 leads to phosphorylation of either ERK2 or c-Jun N-terminal kinase 1 (JNK1), and polarization of the MTOC with cytolytic granules (Chen et al., 2007). Additional signaling molecules linked by PI3K signaling, downstream to NKG2D, include the Crk-like adaptor protein, CrkL, and Ras family GTPase Rap1, which were shown to be important for MTOC polarization and cytotoxicity (Segovis et al., 2009). In contrast to the process of MTOC reorientation, the convergence of lytic granules to the MTOC depends on early upstream Src kinase signaling (James et al., 2013).

MTOC polarization to the NKIS is thus intimately associated with and dependent on cytoskeletal reorganization (Orange et al., 2002, 2003; Graham et al., 2006; Butler and Cooper, 2009). Therefore mediatory molecules are probably involved in cytoskeletal and microtubule dynamics. One of the proteins identified, which links these two networks, is Cdc42-interacting protein-4 (CIP4), which associates with Cdc42 and WASp (Banerjee et al., 2007). CIP4 links the actin and microtubule cytoskeletons in activated NK cells, facilitating MTOC polarization and NK cell cytotoxicity. It is possible that additional mediating molecules such as CIP4 function to merge these two cytoskeletal networks, and it would be interesting to investigate how these are differentially regulated under inhibitory and activating conditions. Moreover, it is possible that decoupling of the actin and microtubule cytoskeletons promotes NK tolerance. It is interesting to speculate that abrogation of MTOC association with the actin cytoskeleton may also lead to dysfunction in primary immunodeficiency and other chronic diseases.

Interestingly, additional functions mediated by microtubule dynamics have been suggested in T-cells. For example, TCR micro clusters were shown to localize at the IS on microtubules through Myosin II and Dynein motors (Hashimoto-tane et al., 2011). Disruption of MTOC polarization to the T-cell IS through Dynein inhibition reduced phosphorylation of ZAP70, LAT, and VAV1, and caused the creation of a malformed T-cell IS characterized by low accumulation of CD3 in the center of the synapse, and low accumulation of LFA-1 at its periphery (Martín-Cófreces et al., 2008). Furthermore, inhibition of the microtubule end binding protein EB1 abrogated LAT/PLCγ-1 complex association and subsequent TCR activation signaling (Martín-Cófreces et al., 2012). This raises the question of whether the MTOC has an additional function as a scaffold to deliver further signaling molecules to the IS, thereby enabling correct signaling cascades, and whether microtubule dynamics, similar to F-actin flow, also play a role in regulating activation signaling. Just as F-actin retrograde flow was seen to impact and sustain correct PLCγ-1 activity in T-cells (Babich et al., 2012), microtubule dynamics may also play a role in influencing sustained T-cell signaling, rather than functioning solely as a signaling scaffold and delivering vesicles to the IS. The microtubule cytoskeleton is thus of great importance in controlled NK cell effector function, however, the molecular mechanisms in NK cells that induce MTOC polarity and positioning at the IS have not been extensively explored. It is possible that such signaling circuits are dysregulated during different chronic pathological conditions (i.e., chronic infection and cancer), enabling escape from NK cell immune surveillance.

#### NK Cell-Mediated Killing

After firm adhesion and sufficient activating signaling, the next phase of NK cell function involves reorientation of the MTOC toward the IS, and subsequent release of lytic granules for target cell killing. The actin cytoskeleton plays multiple roles in this cytotoxic phase. As described, the first step in NK cell cytotoxicity requires cytolytic granule convergence onto the MTOC before MTOC polarization to the NKIS (Mentlik et al., 2010). This process is crucial for prevention of bystander cell killing (Hsu et al., 2016). The MTOC subsequently polarizes to the NKIS, and it was shown that F-actin polymerization is vital for this process (Orange et al., 2002; Butler and Cooper, 2009). Following MTOC polarization and anchoring at the IS, lytic granules move rapidly across the dense F-actin network at the cell membrane prior to degranulation; in order to ensure persistent degranulation, the actin meshwork must remain intact (Mace et al., 2012). Lytic granules associate with Myosin IIA, and this association is believed to coordinate with the F-actin cytoskeleton for lytic granule delivery to the IS, and to provide physical forces to “squeeze” granules through the actin meshwork. Abrogation of NM-IIA activity using inhibitors or site directed mutations reduces the ability of lytic granules to bind to F-actin, and impedes granule entry into the actin meshwork at the IS (Andzelm et al., 2007; Sanborn et al., 2009). Due to the accumulation of a dense actin network at the IS prior to degranulation, the mechanism that enables escape of the lytic granule content of the NK cell is difficult to resolve. Advanced microscopic techniques from the Davis and Orange groups elucidated the mechanisms of lytic granule secretion across the actin boundary: lytic granules traverse the dense actin network at the IS until reaching, and docking at specific areas with low actin density, from where they can be released toward the target cell (Brown et al., 2011; Rak et al., 2011). Disrupting NK cell actin dynamics immediately prior to degranulation inhibited granule release (Rak et al., 2011). In addition to lytic granule secretion, a different Myosin independent mechanism was shown to enable cytokine secretion at the NKIS, and this was similarly dependent on formation of local actin pores at the NKIS (Brown et al., 2012). As mentioned earlier, one mediator of actin clearance at the NKIS is Coronin1A, which is essential for generating precisely targeted actin clearances by promoting localized actin depolymerization (Mace and Orange, 2014). Therefore, meticulous regulation of localized actin disassembly enables precise delivery of granules across the synaptic cleft. This mechanism most probably ensures selective delivery to target cells while avoiding lytic granule spillage that can affect bystander tissue. It is not yet known how Coronin1A is regulated during stimulation of NK cells, and how its activity is coupled directly with granule exocytosis. It is possible that generation of actin clearances occur in a stochastic fashion, followed by random movement of granules across the actin network until they reach low actin areas. It would be interesting to resolve the mechanisms and mediators of Coronin1A recruitment to the IS, and how localized actin deconstruction harmonizes with the additional actin architectures present at the IS. For example, Carisey et al. (2018) recently described additional actin structures that are critical for NK cell cytotoxicity. Local actin dynamic puncta are generated via Arp2/3 and NM-IIA activity at the NKIS, and these structures are required for lytic granule exocytosis (Carisey et al., 2018). It is possible that localized actin dynamics promote additional forces for delivery of granules, or provide additional motion that resonates along the synaptic actin sheet that enhances the possibility of lytic granule arrival to areas of low actin content. Further experiments could elucidate how these structures are regulated by different early upstream signaling complexes and NPFs, and in the context of different NK cell receptor ligations.

## The Cytoskeleton in NK Cell-Related Pathologies

Due to the important and variegated roles the cytoskeleton plays in NK cell function, it is not surprising that various immune-related diseases result from cytoskeletal mis-regulation in NK cells (Lagrue et al., 2013; Ham and Billadeau, 2014) (Table 1). Two of the most well characterized immunodeficiencies are WAS/X-linked thrombocytopenia (XLT). WAS is an X-linked immunodeficiency characterized by mutations that have varying effects on WASp expression. Different phenotypes are caused by a complete or partial absence of WASp expression in affected patients (Derry et al., 1994). WAS patients with complete WASp depletion suffer from reduced platelet count, complications in blood clotting, eczema, recurrent infections and cancer (Sullivan et al., 1994). A less severe form of WAS known as XLT occurs due partial WASp expression resulting mainly in microthrombocytopenia (Imai et al., 2004). It was demonstrated that NK cells from healthy donors express high levels of WASp, while NK cells from WAS patients express no detectable levels (Orange et al., 2002). Given the importance of WASp in actin regulation, it is not surprising that mutations in the protein or its degradation have severe impacts. As mentioned earlier, the cytoskeleton plays a paramount role in leukocyte migration, as the actin machinery propels the cell and changes its morphology in order to navigate through blood and tissue (Vicente-Manzanares and Sánchez-Madrid, 2004). WASp deficiency in NK cells severely damages their migratory capabilities (Stabile et al., 2010). Both WAS and XLT NK cells exposed to the migration-inducing cytokines CXCL12/SDF-1 or CX3CL1/fractalkine and placed on adhesion molecule (ICAM-1/VCAM-1) coated filters show low cellular migration compared to wild-type NK cells (Stabile et al., 2010). NK cells from WASp-deficient mice exhibit defects in tumor suppression (Catucci et al., 2014), and a significantly reduced cytotoxic potential relative to healthy NK cells (Orange et al., 2002). This is predominantly due to lower actin accumulation at the NKIS that impacts synaptic clustering of activating receptors (Orange et al., 2002; Gismondi et al., 2004). As mentioned earlier, it was suggested -that bypassing WASp deficiency in NK cells might be enabled via IL-2 administration, leading to actin reorganization via WAVE2 (Orange et al., 2011).

**TABLE 1:** Diseases affecting the NK cell cytoskeletal machinery.

**Disease**	**Defect in protein**	**Phenotype**
Wiskott–Aldrich Syndrome (WAS)/X-linked thrombocytopenia (XLT)	Partial or no expression of WASp	Patients display low blood platelet count, deficiency in blood clotting, recurrent infections and eczema. NK cells exhibit deficiency in migration, and low accumulation of F-actin at the NKIS resulting in diminished NK cell activation and cytotoxicity.
WASP-interacting protein (WIP) deficiency	No expression of WIP	Patient displayed recurrent infections, eczema, thrombocytopenia, and defective T cell proliferation and chemotaxis. NK cells demonstrated defective effector function.
Dedicator of cytokinesis 8 (DOCK8) deficiency	No expression of DOCK8	Patients display immunodeficiency characterized by sino-pulmonary and cutaneous viral infections. NK cells are impaired in IS formation due to reduction in F-actin accumulation, leading to inhibited cytolytic function.
Dedicator of cytokinesis 2 (DOCK2) deficiency	No expression of DOCK2	Patients display immunodeficiency characterized by recurrent infections, lymphopenia, and thrombocytopenia. NK cells are impaired in effector functions such as degranulation and cytokine secretion due to abrogated F-actin polymerization.
RAS guanyl-releasing protein 1 (RASGRP1) deficiency	No expression of RASGRP1	Patient displayed primary immunodeficiency disorder, characterized by recurring infections, and ultimately, development of low-grade Epstein–Barr virus (EBV)-associated B cell lymphoma. NK cells are deficient in F-actin accumulation, lytic granule convergence to the MTOC, and degranulation.
Chediak–Higashi syndrome	Mutations in the regulator of lysosomal trafficking, LYST	Patients display dysregulated immune function, albinism, predisposition to bleeding, and hyper inflammation. NK cells with LYST mutations contain enlarged lytic granules that cannot penetrate the actin mesh at the NKIS, impairing degranulation.
Coronin 1A deficiency	No expression of Coronin 1A	Patients display immunodeficiency characterized by recurrent viral infections and lymphopenia. NK cells demonstrate abrogated deconstruction of F-actin at the IS, impeding lytic granule secretion and thus, cytotoxicity.
Hermansky–Pudlak syndrome type 2	Mutations in the β3A subunit of the AP-3 adaptor protein	Patients display albinism, recurrent infections, susceptibility to bleeding, and hemophagocytic lymphohistiocytosis. NK cells are defective in degranulation due to formation of enlarged lytic granules.
Myosin IIA mutations (May-Hegglin anomaly, Sebastian syndrome, Fechtner syndrome, and Epstein syndrome)	MYH9 gene mutation	Patients present macrothrombocytopenia, leukocyte inclusion bodies, low platelet count and recurrent bleeding. Patients also display hearing loss, nephropathy, and cataracts. NK cells are characterized by impaired cytotoxicity.
Griscelli syndrome type II	Mutation in RAB27A	Patients display hypopigmentation, immunodeficiency, and hemophagocytic lymphohistiocytosis. NK cells display impaired cytotoxicity due to docking failure of granules at the plasma membrane.
Familial Hemophagocytic Lymphohistiocytosis Types 2–5	Mutations in perforin 1 (FHL type 2), Munc13-4 (FHL type 3), syntaxin 11 (FHL type 4), and Munc18-2 (FHL type 5)	Patients display immune dysregulation characterized by hyper inflammation, fever, enlarged spleen and liver, and hemophagocytosis. NK cell degranulation and/or cytotoxicity is severely impaired.

WASp-interacting protein deficiency leads to a reduction in NK cell functional output (Noy et al., 2012). WIP helps to protect WASp from ubiquitin-mediated degradation (Fried et al., 2014b), but also has additional functions in NK cells. As described earlier, following NK activation, WIP mediates assembly of a protein complex comprising WASp, actin, and NM-IIA (Krzewski et al., 2006). In addition, WIP was also found to be essential for granule-mediated exocytosis in NK cells, since it associates with lytic granules in NK cells, and its depletion from NK cells causes a failure in lytic granule polarization (Krzewski et al., 2008). A female patient bearing a mutation containing a stop codon in the *WIPF1* gene, which encodes WIP, displayed recurrent infections, eczema, thrombocytopenia, defective T cell proliferation and chemotaxis, and impaired NK cell effector function (Lanzi et al., 2012).

Another disease impacting F-actin organization at the NKIS is DOCK8 deficiency. As described earlier, DOCK8 belongs to the superfamily of DOCK180 GEFs for the Rho protein family (such as Cdc42) (Côté and Vuori, 2002; Sinai et al., 2010; Stabile et al., 2010). NK cells from patients with DOCK8 deficiency are not able to form a mature IS due to reduced F-actin accumulation. This results in a decrease in cytotoxicity that cannot be bypassed by IL-2 administration, and could explain why patients with DOCK8 deficiency are susceptible to sino-pulmonary and cutaneous viral infections (Zhang et al., 2009; Mizesko et al., 2013). Hence, unlike WASp deficient NK cells, whose function might be recovered through IL-2 mediated activation of WAVE2, DOCK8 seems to be indispensable for proper actin accumulation at the NKIS. Additional work is expected to unravel the molecular mechanisms connecting DOCK8 to the activating or inhibiting NKIS. Similarly to DOCK8 deficiency, inherited DOCK2 mutations in five patients with recurrent bacterial and viral infections and lymphopenia were shown to impact T/B cell and NK cell responses (Dobbs et al., 2015). NK cells in these patients show reduced migration and actin polymerization, as well as impaired degranulation.

A role for RASGRP1 was also demonstrated in an NK cell immunodeficiency (Salzer et al., 2016). As mentioned earlier, RASGRP1 acts as a GEF for Ras, thereby activating the Ras pathway and the MAPK cascade (Downward et al., 1990; Roose and Weiss, 2000; Stone et al., 2000). A patient with RASGRP1 deficiency displayed a primary immunodeficiency disorder, characterized by recurring infections, and ultimately developed low-grade Epstein–Barr virus (EBV)-associated B cell lymphoma (Salzer et al., 2016). NK cells from patients with RASGRP1 deficiency do not form a mature NKIS, and their IS is characterized by decreased actin accumulation and polarization of the MTOC, and accordingly, a lower capacity for cytotoxicity toward target cells (Salzer et al., 2016). These phenotypes might arise due to the importance of the MAPK pathway in NK cell actin rearrangement (Vély and Vivier, 2005). Interestingly the same study reported association of RASGRP1 with Dynein light chain DYNLL1. Imaging NK cells from this patient revealed defective granule motility, and because Dynein is important for convergence of lytic granules onto the MTOC, this may account for an additional factor inducing lower NK cell-mediated cytotoxicity (Mentlik et al., 2010; Salzer et al., 2016).

Highlighting another aspect of cytoskeletal regulation, the inability to properly clear the actin meshwork at the NKIS for granule secretion in NK cells was recently described in Chediak–Higashi syndrome (Gil-Krzewska et al., 2017). This disease is caused by mutations in the regulator of lysosomal trafficking, LYST (Nagle et al., 1996), and is characterized by hyper inflammation and impaired functionality of CD8 T and NK cells (Introne et al., 1999; Karim et al., 2002; Lozano et al., 2014). NK cells with impaired LYST function contain enlarged granules, and thus, the NKIS effectively acts as a barrier for exocytosis. Use of the actin inhibitors latrunculin A or swinholide A increases the permeability of the actin mesh and restores secretion from these NK cells (Gil-Krzewska et al., 2017). This study further illustrates that actin disassembly is also critical in maintaining proper cytolytic function. In addition to Chediak–Higashi syndrome, Hermansky–Pudlak syndrome type 2 also causes formation of enlarged granules, induced by mutations in the β3A subunit of AP-3 (Dell’Angelica et al., 1999). AP-3 is an adaptor protein that interacts with the clathrin scaffold protein, facilitating sorting of proteins to lysosomes (Dell’Angelica et al., 1997). NK cells from patients with Hermansky–Pudlak syndrome type 2 also show disrupted effector functions (Fontana et al., 2006). It is possible that this disorder also disrupts secretion of enlarged lytic granules at the NKIS, but additional studies are required to verify this mechanism.

Mutations in the heavy chain of Myosin IIA also lead to a variety of diseases such as May-Hegglin anomaly, Sebastian syndrome, Fechtner syndrome, and Epstein syndrome, characterized by macrothrombocytopenia with leukocyte inclusions (Seri et al., 2003). As discussed, NM-IIA is important for delivery of cytolytic granules through the NKIS (Andzelm et al., 2007). Several mutations that alter normative NM-IIA conformation impact its regulation of cytotoxicity in NK cells. For example, a mutation in May-Hegglin anomaly patients with a C-terminal truncation of MYH9 at position 1933 causes a reduction in NK cell cytotoxicity (Sanborn et al., 2009). Furthermore, Sanborn et al. (2011) mapped various mutations in NM-IIA which cause similar phenotypes of reduced NK cell cytotoxicity. For example, a S96L mutation in the head region and T1155I mutation in the S2 region result in a decrease in NK cell killing. A truncation of the protein at residue 1942, which is located on the tailpiece, also causes a reduction in NK cell killing (Sanborn et al., 2011). Interestingly, the same study showed that phosphorylation of the NM-IIA tailpiece at S1943 is critical for Myosin function at the NKIS; hence, mutations in this regulatory area may account for the phenotypes observed (Sanborn et al., 2011). It is also possible that additional activity of Myosin at the NKIS influences the dysfunction of NK cell cytotoxicity. As mentioned previously, NM-IIA forms a complex with actin, WIP, and WASp during NK cell activation (Krzewski et al., 2006), and it is present in the pSMAC with actin filaments in acto-Myosin arcs. Therefore, NM-IIA may also drive IS formation and stability which are restricted in NM-IIA-related diseases (Hammer and Burkhardt, 2013).

Other diseases that impact lytic granule and cytoskeletal cross-talk in NK cells include Griscelli syndrome type II and Familial Hemophagocytic Lymphohistiocytosis (FHL) Types 2-5 (Ham and Billadeau, 2014). Griscelli syndrome type II is caused by mutation in RAB27A, a member of the small GTPase family (Ménasché et al., 2000). RAB27A was shown to play a role in cytoskeletal dependent lytic granule movement in the plasma membrane and cytosol of NK cells (Liu et al., 2010), possibly through a complex with the motor protein Kinesin-1 and synaptotagmin-like protein 3 (Kurowska et al., 2012). NK cells from Griscelli syndrome type II patients display impaired cytotoxicity due to docking failure at the plasma membrane (Wood et al., 2009). FHL Type 3 is caused by mutations in Munc13-4 (Feldmann et al., 2003), which is involved in vesicle priming. Mutations in Munc13-4 that abrogate its association to RAB27A inhibit degranulation in cytotoxic T-cells (Elstak et al., 2011), and NK cells deficient in Mucn13-4 are inhibited in granule secretion (Wood et al., 2009). Importantly, recruitment of Rab27a and Munc13-4 to lytic granules is Myosin-dependent (Wood et al., 2009), further emphasizing the role of cytoskeletal compartments in effector NK cell responses. Additional FHL diseases are caused by different mutations. FHL type 2 is caused by mutations in the perforin 1 gene (Stepp et al., 1999), abrogating the ability of NK cells to lyse target cells (Marcenaro et al., 2006). FHL type 4 is caused by mutations in syntaxin 11 (Bryceson et al., 2007), inhibiting the ability of NK cells to degranulate. Finally, FHL 5 is caused by mutations in Munc18-2, which also severely impairs NK cell exocytosis (Côte et al., 2009).

Due to the great importance of NK cells in innate immunity, it is not surprising that various conditions result from functional NK cell deficiency (FNKD) syndromes that may arise from defects in the NK cytoskeleton, such as Herpesvirus infection, multiple infections, presence of intracellular bacteria, and Human Papillomavirus (HPV) (Orange, 2013). The protective effect of NK cell immune surveillance on cancer in humans has been documented (Imai et al., 2000; Ishigami et al., 2000; Villegas et al., 2002), and this is especially evident in the outcome of patients who were administered NK cells from donors that have the advantage of having graft versus leukemia activity in the recipient without causing graft versus host disease (Hsu et al., 2005). It is not known whether tumor growth is increased on the background of FNKD syndromes that involve the NK cytoskeleton. Future studies could reveal if functional dysregulation of NK cell cytoskeletal activity may promote other diseases or malignancies, and thus prompt development of therapies to bolster NK activity.

## NK Cell Inhibitory Signaling and Cytoskeletal Dynamics at the NKIS

Inhibition of NK cells does not occur independently on its own, that is, without input from additional activating receptors on the NK cell surface. Co-engagement of inhibitory receptors with activation receptors prevents NK cell activation; therefore, suppression of NK cell activity by inhibitory receptors can be thought of as co-inhibition (Long et al., 2013). Photo stimulation of the inhibitory killer-cell immunoglobulin-like receptor (KIR) KIR2DL2 during ongoing NK cell activation is not sufficient to prevent calcium flux, however, it induces rapid formation of inhibitory microclusters that prevent formation of activating clusters and promote retraction of the NK cell (Abeyweera et al., 2011). Therefore, inhibitory receptor signaling prevents activation of NK cells from manifesting in the first place. Inhibitory NK cell signaling involves dephosphorylation and/or degradation of upstream signaling proteins and dismantling of activating signaling complexes (Long, 2008; Peterson and Long, 2008; Watzl and Long, 2010). Accordingly, NK cell inhibition has substantial effects on actin polymerization and rearrangement. NK cells express inhibitory receptors that contain immunoreceptor tyrosine based inhibition motifs (ITIMs) in their cytoplasmic tails that bind to several Human Leukocyte Antigen (HLA) isoforms. The best defined of these receptors in humans are KIRs and NKG2A/CD94 (Wagtmann et al., 1995; Moretta et al., 2002). Engagement of inhibitory receptors with their cognate ligands results in phosphorylation of the ITIM motifs, and it has been suggested that the phosphorylation is carried out by the Src family kinases (Long, 2008). Phosphorylation on ITIMs prompts recruitment of SHIP-1 or SH2-domain-containing protein tyrosine phosphatase (SHP-1/2) (Long, 1999; Ravetch, 2000; Purdy and Campbell, 2009), which induce de-phosphorylation of downstream signaling molecules important for NK activation (Long, 2008).

Killer-cell immunoglobulin-like receptor receptors may not require actin reorganization for their recruitment to the NKIS (Davis et al., 1999). Work by Stebbins et al. (2003) using a SHP-1 trapping mutant that is catalytically inactive but capable of binding phosphorylated substrates detected VAV1 as the first verified SHP-1 substrate in NK cells. The same study also demonstrated that the process occurs independently of actin rearrangement, as dephosphorylation of VAV1 occurred in the presence of actin inhibitors (Stebbins et al., 2003). VAV1 activity may also be regulated by the E3 ubiquitin ligase c-Cbl. Cooperative activation of the activating NKG2D and 2B4 receptors is necessary to circumvent inhibition of VAV1 by c-Cbl (Kim et al., 2010). An additional inhibitory mechanism independent of VAV1 was suggested to operate through the Crk adaptor protein, which is involved in cytoskeletal remodeling (Antoku and Mayer, 2009). Crk is phosphorylated and associates with c-Abl upon clustering of inhibitory receptors (Peterson and Long, 2008). We previously demonstrated that PLCγ-1/2 and LAT are also dephosphorylated and inactivated by SHP-1 during NK cell inhibition, and that ubiquitination of LAT by c-Cbl and Cbl-b serves as an additional mechanism to ensure tolerance, by sequestering remaining phosphorylated LAT from the NKIS (Matalon et al., 2016). Therefore, it appears that NK cell inhibition involves multiple modules. These include, on the one hand, blocking substantial F-actin reorganization at the IS through dephosphorylation of VAV1 and phosphorylation of Crk, and on the other hand, inhibiting formation of activating signaling complexes such as PLCγ and LAT to possibly prevent formation of secondary messengers (i.e., IP3 and DAG), early activation, and calcium flux.

### The Inhibitory NKIS

The inhibitory NKIS is characterized by disorganized molecular segregation, instability, and short lifetime. These inhibitory characteristics prevent NK cell activation and effector outcome, serving as a key checkpoint in regulating cytotoxicity and maintaining tolerance (Davis et al., 1999).

Due to the activity of VAV1 in actin polymerization and rearrangement through the Rac pathway, it is expected that its inactivation would hamper various actin-dependent processes. F-actin accumulation and density are much greater in synapses of NK cells that are exposed to susceptible activating targets, than on targets that induce an inhibitory NKIS (Banerjee and Orange, 2010). As mentioned earlier, actin accumulation and lipid raft recruitment to the NKIS were shown to be disrupted upon NKG2A/CD94 receptor-mediated inhibition, when SHP-1 is recruited to the NKIS and VAV1 phosphorylation levels are decreased (Masilamani et al., 2006). Accumulation of 2B4 and NKG2D activating receptors is actin dependent, and this clustering is also abrogated during KIR receptor-HLA binding (Watzl and Long, 2003). Inhibition via KIR2DL2 additionally inhibits activating receptor clustering, and reduces NK cell spreading via SHP1/2 activity (Abeyweera et al., 2011). Thus, NK cells developed mechanisms to first avoid reactivity by differential regulation of cytoskeletal dynamics. Due to the dependence of NK activation on accumulated actin, which recruits signaling clusters to the NKIS, regulation of actin assembly at the NKIS ensures tolerance. It is not clear whether VAV-1 dephosphorylation through SHP-1 and phosphorylation of Crk are the sole mechanisms for regulating actin dynamics at the NKIS, and what other factors, if any, sequester F-actin mediated activation at the NKIS to prevent normal synapse formation and activation. For example, Kopcow et al. (2005) showed that decidual NK cells (dNKs) do polarize actin toward their synapse; however, the synapse remains inert as the MTOC does not polarize (Kopcow et al., 2005). Hence, additional inhibitory mechanisms that regulate cytotoxicity through maintenance of the cytoskeleton, possibly linking cytoskeletal reorganization with MTOC polarization, should be investigated. It is tempting to speculate that molecules such as CIP4, which link the microtubule and actin cytoskeletons, may be regulated or expressed differently in decidual NK cell subtypes.

The inhibitory IS favors accumulation of inhibitory KIRs into subdomains coined supra-molecular inhibition clusters (SMICs) (Davis et al., 1999). Phosphorylated KIR receptors form microclusters with the tyrosine kinase, Lck, during NK cell inhibition (Treanor et al., 2006). The SMIC of the inhibiting NKIS contains a different composition of signaling molecules, including phosphatases such as SHP-1 (Vyas et al., 2002a) and SHP-2 (Purdy and Campbell, 2009). The accumulation of inhibiting signaling molecules in the SMIC disrupts the stability of the IS, leading to detachment from the target cell, and favoring migration (Burshtyn et al., 2000; Masilamani et al., 2006). The inhibitory NKIS differs from the activating NKIS, as it is not radially symmetrical, and not stable. A similar “make and break” synapse has been characterized in T-cells, and coined the kinapse. Similarly to the kinapse, the inhibitory NKIS is characterized by smaller size and much lower NK:target cell conjugation times (Burshtyn et al., 2000; Sims et al., 2007; Culley et al., 2009).

Unlike activating synapses, the accumulation of KIRs does not causeextensive accumulation and rearrangement of actin (McCann et al., 2003). The recruitment of KIRs to the NKIS and their phosphorylation appears to be actin independent and occurs upstream to actin rearrangement; however, this issue is not yet resolved (Faure et al., 2003). An observable mesh of cortical actin seems unchanged at inhibitory synapses relative to activating synapses, yet these synapses seem to lack a dense peripheral actin ring (Brown et al., 2011). Fassett et al. (2001) showed that actin polymerization is crucial for lipid raft polarization in inhibitory NK cell synapses. In addition, using the inhibitor of actin polymerization, cytochalasin D, Standeven et al. (2004) demonstrated that actin dynamics may be important in early KIR2DL1 recruitment to the inhibitory NKIS. In NK cells engaged with multiple targets, KIR2DL1 was also shown to relocate between different synapses (Pageon et al., 2013a), and coalescence of KIRs into micro-clusters was shown to depend on cytoskeletal dynamics (Pageon et al., 2013b). Moreover, during NK cell inhibition, large KIR2DL1 clusters seem to favor the phosphorylation and activation of SHP-1 (Oszmiana et al., 2016). In this study, the localization of the activating KIR, KIR2DS1 in signaling clusters depended on the lysine 233 residue in the transmembrane sequence; yet, it is not clear if cluster size of the inhibitory and activating KIRs was also influenced by different cytoskeletal reorganization modes. Interestingly, a recent study from the Davis group investigated how KIR variation impacts organization on the cell membrane (Kennedy et al., 2019). NK cells were divided into groups with either low or high surface expression of KIRs. Higher receptor abundance correlated with increased cluster size, yet with more receptors observed outside clusters. On the other hand, cells expressing low surface abundance of KIRs appear to dominantly coalesce into clusters and to favor increased phosphorylation of Crk. Interestingly, it appears that since SHP-1 activity is favored at large KIR clusters, its activity may promote dephosphorylation of Crk and prime NK cells that express more inhibitory receptors on the cell membrane (Kennedy et al., 2019). Thus, the organization of inhibitory signaling molecules on the NK cell surface has critical ramifications for the basal NK cell activation state. How and why different KIR genotypes are expressed at different concentrations and coalesce differently into signaling clusters remains to be determined. The role of the cytoskeleton in mediating this organization is an additional intriguing area of study. As discussed in the following sections, it is possible that cytoskeleton-mediated reorganization of receptors on the NK cell membrane during development may dictate their activation threshold.

## The Effect of the Target Cell Cytoskeleton on NK Cell Function

In addition to the vast importance of cytoskeletal rearrangement on NK cell function, it is becoming increasingly evident that the cytoskeletal architecture on target cells can greatly influence NK cell effector efficacy. T-cells and B-cells are usually activated to a greater degree when confronted with stiffer *in vitro* substrates (as shown using coated slides with varying rigidities) and target cells which display stiffer properties (stiffer cortical actin and more actin stress fiber assembly) (Huse, 2017; Saitakis et al., 2017; Shaheen et al., 2017; Ben-Shmuel et al., 2019). Moreover, it is clear that constraining target cell co-stimulating ligands (such as ICAM-1) by the target cell cytoskeleton, induces higher T-cell activation (Comrie et al., 2015b; Comrie and Burkhardt, 2016). This may be due to counter force provided by the constrained ligand to receptors on T-lymphocytes. Therefore, strategies that target immune cell cytoskeletal machinery must also take into consideration the possible effects on the integrity of the target cell cytoskeleton, which impact lymphocyte activation.

In the case of NK cells, it was previously demonstrated that similar mechanisms operate on target cells to modulate the NK cell responses. Gross et al. (2010) showed that impairing cytoskeletal integrity by treating target cells with Latrunculin A (which disrupts microfilament organization by binding to monomeric G-actin) leads to inhibition of NK cell LFA-1 mediated conjugation and polarization of lytic granules to the NKIS; these effects were caused by higher mobility of ICAM-1/2 on target cells due to compromised association with the cytoskeleton. Furthermore, actin polymerization was shown to be crucial for MHC class I recruitment in dendritic cell (DCs) targets during their interaction with NK cells, resulting in KIR engagement and inhibition of NK cells. Inhibition of actin in DCs results in increased IFNγ secretion (Barreira da Silva et al., 2011).

Recently, work by Al Absi et al. (2018) demonstrated that cytoskeletal remodeling in tumor targets can have drastic ramifications on NK cell cancer surveillance. In this work, breast adenocarcinoma cells were shown to initiate an “actin-response” to NK cell conjugation. This response is characterized by substantial amounts of actin accumulation by the cancer cell at the NKIS. Actin accumulation significantly protected the cancer cell from NK-mediated cytotoxicity. One possible explanation for escape from cell death is generation of a physical barrier that is not permissive for lytic granules. Moreover, the actin response in this study correlated with the epithelial to mesenchymal transition of the breast cancer cells, and interestingly, it induced the coalescence of HLA ligands and PD-L1 at the NKIS, possibly shedding some light on an additional mechanism of escape from NK cell surveillance, through clustering of inhibitory checkpoint ligands.

In addition to this mechanism in cancer cells, previous work demonstrated that alterations of cytoskeletal architecture through viral infection can also down modulate NK cell effector function. Stanton et al. (2014) showed that target cells infected with the HCMV pUL135 strain have dramatically altered cytoskeletal morphology, characterized by cell rounding, loss of focal adhesions, loss of actin stress fibers, loss of cell projections, and an increase in cortical actin. These features severely inhibited NK cell killing of target cells, specifically due to impaired conjugation and IS formation. It is tempting to speculate that these changes in target cell morphology decrease the mechanical characteristics of the target cell, which were shown to significantly impact the potentiation of cytotoxic T-cells (Basu et al., 2016).

A question that can be raised is how can NK cell effector function be modulated through targeting actin dynamics? In the study by Al Absi et al. (2018), targeting actin nucleation factors such as N-WASp or Cdc42 in target cells restored NK cell mediated cytotoxicity by inhibiting the cancer cell actin response. We also previously showed that the centripetal flow of actin at the inhibitory NKIS may affect the enzymatic activity of SHP-1 (Matalon et al., 2018). Interestingly, NK cells stimulated on stiff substrates are characterized by extremely reduced centripetal flow, which may constrain SHP-1 activity, inducing chronic NK cell activation (Matalon et al., 2018). Targeting NPFs to modulate inhibitory checkpoint signaling may therefore provide an additional strategy to modify NK cell activity. Furthermore, work by the Davis group examined the effect of the drug Lenalidomide on NK cell effector function against multiple myeloma. Lenalidomide enhanced NK cell effector function by reducing the periodicity of the actin mesh at the NKIS, facilitating lytic granule and cytokine secretion (Lagrue et al., 2015). In addition to this study, Lagrue et al. (2015) showed that treating B-cells with the monoclonal antibody Rituximab, which binds CD20, results in recruitment of ICAM-1, moesin [a member of the ERM family of proteins that links the cortical cytoskeleton to the plasma membrane (Tsukita and Yonemura, 1999)], and CD45 to the CD20 cap (Rudnicka et al., 2013). Rituximab also induced MTOC polarization in B-cells. The polarization events induced by rituximab treatment significantly increased NK cell mediated ADCC. It is also possible that accumulation of adhesion molecules, ERM proteins, and cytoskeletal components at the membrane of target cells treated with rituximab provides a firm substrate that more efficiently activates cytotoxic lymphocytes. These data support findings that the cell tension of target cells significantly potentiates cytotoxic T-cell activity (Basu et al., 2016). Therefore, development of treatments that can also modulate the physical properties of target cells at the interface with cytotoxic lymphocytes may be interesting to pursue.

## The Road Ahead – Unlocking the Reciprocal Role of the Acto-Myosin Machinery on Lymphocyte Signaling

There are several studies that raise the exciting possibility of acto-Myosin dynamics directly influencing signaling in lymphocytes, possibly through processes of mechanotransduction (Huse, 2017; Ben-Shmuel et al., 2019). Work from the Burkhardt lab showed correlations between centripetal actin flow and levels of tyrosine phosphorylation profiles of critical cytoplasmic signaling molecules. A study by Babich et al. (2012) demonstrated that actin retrograde flow sustains T-cell signaling through PLCγ1, leading to T-cell calcium flux and activation. Interestingly, the same study demonstrated that ZAP-70 kinase phosphorylation remained intact despite perturbations in actin retrograde flow, demonstrating a selectivity that may be orchestrated by actin remodeling. Furthermore, SLP-76 seems to be uncoupled from actin dynamics, moving centrally at a faster velocity than the observed ARF in the lamellum and cell body, raising the possibility of differential regulation of signaling molecules and clusters by the cytoskeleton. This may indicate that centralization of SLP-76 relies on additional factors, possibly other cytoskeletal components such as the microtubule network. Another work by Jankowska et al. (2018) conducted in T-cells showed that after LFA-1 and VLA-4 integrins engage with cognate ligands, there is a dampening of actin retrograde flow associated with abrogation of tyrosine phosphorylation downstream to TCR engagement. Hence it is evident that there exists a complex regulatory mechanism controlled through actin movement, in addition to the roles of the cytoskeleton in assembly of signaling complexes. An interesting possibility raised by the results of Jankowska et al. (2018) is the induction of inhibitory tyrosine phosphatase activity by cytoskeletal dynamics and substrate mechanics. For example, we previously showed that F-actin dynamics at the inhibitory NKIS may regulate the enzymatic activity of SHP-1 (Matalon et al., 2018). Reduced F-actin dynamics may promote SHP-1 binding to the cytoskeleton and release it from auto-inhibition. Thus, by controlling the phosphorylation and activation profile of key signaling molecules through phosphatase function, the cytoskeleton could potentially facilitate rapid differentiation between activating and inhibiting target cells. Local actin reorganization at an activating synapse may sequester SHP-1 pools, enabling their simultaneous assembly and activity at inhibitory synapses. Another possibility is that specific pools of SHP-1 that are recruited independently of cytoskeletal rearrangement to ITIM domains decrease cytoskeletal turnover at inhibitory synapses, subsequently promoting the association of additional SHP-1 molecules to the cytoskeleton. Such a mechanism may promote assembly of SHP-1: substrate complexes in a positive feedback process promoting inhibition.

Not much is known regarding how cytoskeletal assembly can influence NK cell signaling intermediates, beyond cytoskeletal function at the NKIS. As discussed earlier, the assortment of signaling receptors at the NKIS is instrumental in determining NK cell activity. One outcome of cytoskeletal regulation of surface receptors on NK cells may be tuning of NK cell responses in the context of education. NK cell education refers to the process wherein NK cells are quantitatively primed by MHC-I molecules during development and acquire the capacity to elicit effector function. Guia et al. (2011) demonstrated that the distribution of NK cell activating/inhibitory receptors confined by the actin meshwork controls NK cell education. NK cell education is believed to be primarily regulated by NK cell exposure to inhibiting ligands, and depends on the strength and number of NK cell inhibiting receptors and target cell ligands (Orr and Lanier, 2010). Guia et al. (2011) showed that activating and inhibitory receptors are confined together by the actin cytoskeleton in hyporesponsive NK cells. Thus, the actin cytoskeleton may abrogate activating signaling by organizing activating receptors in the vicinity of inhibitory receptors. In responsive NK cells, on the other hand, activating receptors localize in nanodomains, thereby promoting favorable signaling, whereas inhibiting receptors are confined in the actin meshwork. Interestingly, Guia et al. (2011) found no significant differences in the NK transcriptional program between educated and non-educated cells. Thus, it remains unclear how ITIM bearing receptors mediate the formation of this intricate architecture through cytoskeletal regulation. Inhibition of the cytoskeleton in this study diminished the confinement of NKp46 in hypo-responsive NK cells; therefore, actin regulatory proteins may differentially orchestrate receptor architecture, and these cues might derive from ITIM signaling intermediaries such as SHP-1/2. As mentioned earlier, higher expression and clustering of KIR molecules favor the activation of SHP-1 which may dephosphorylate and activate Crk (Kennedy et al., 2019). It is interesting to consider that SHP-1-based signaling, which was shown to regulate NK cell education (Viant et al., 2014), could therefore mediate organization of receptors on educated cells through effects on cytoskeletal regulators such as Crk.

It would also be interesting to explore whether other elements that are involved in cytoskeletal regulation differ between the subsets of educated and uneducated NK cells. For example, moesin and α-actinin-1 control ICAM mobility in dendritic cells and their expression increases in mature DCs (Comrie et al., 2015b). It is possible that changes in the expression levels of moesin and α-actinin-1 occur in NK cells throughout their development, controlling NK cell education. This is in line with studies that demonstrated effects of integrins on NK cell education (Enqvist et al., 2015). Specifically, Enqvist et al. (2015) showed that educated NK cells displayed higher concentrations of DNAM-1 and LFA-1 in its high affinity conformation. This implies that the actin network, and perhaps actin dynamics, tune NK cell education by controlling integrin activity. Furthermore, Thomas et al. (2013) showed that inside-out signaling to LFA-1 occurs only in educated NK cells, preserving their cytotoxicity, as opposed to uneducated NK cells that lack proficient inside-out signaling to LFA-1 integrin.

Additional evidence for cytoskeletal regulation of NK cell education was recently shown by Staaf et al. (2018), who demonstrated that the activating NKp46 receptor is more diffuse and the inhibitory LY49A receptor is more confined to microdomains at the surface of educated NK cells. Furthermore, disrupting actin dynamics hampers NKp46 mediated calcium flux in NK cells (Staaf et al., 2018). NKp46 dynamism, mediated by cytoskeletal reorganization, may therefore enhance the probability of engagement with ligands and increase the response of NK cells. Still, it is unclear how education through MHC-I orchestrates these processes. Once again, it is possible that ITIM-dependent signaling primes NK cells through SHP-1 activation of cytoskeletal regulators. This is also evident in studies conducted in T-cells, in which SHP-1 was shown to promote adhesion by dephosphorylation of CrkII (Azoulay-Alfaguter et al., 2017). These are all exciting concepts, as they may open a path to re-sensitization of NK cells through cytoskeletal manipulation.

## Concluding Remarks and Future Perspectives

The cytoskeletal machinery has been established as an indispensable element of NK cell function. NK cells rely on efficient actin and microtubule dynamics, as well as Myosin motor activity for most effector functions. Abnormalities affecting actin turnover, Myosin motor function, and MTOC polarization cause severe impairments in NK cell cytotoxicity and migration, and thereby lead to various pathologies such as primary immune deficiencies.

Multiple ground breaking studies utilizing advanced super resolution microscopy resolved many questions regarding the spatio-temporal organization of the cytoskeleton during NK cell activation and inhibition, and how these different actin morphologies facilitate specific effector functions. These studies also emphasize the importance of receptor and accessory/adhesion molecule architecture on the surface of NK cells, and how it influences the activation threshold and possibly the education state. Furthermore, classic biochemical analyses and assays using specific cytoskeletal inhibitors, or evaluation of NK cells from patients with primary immunodeficiencies enhanced our knowledge regarding key signaling components regulating the cytoskeleton in NK cells.

Important issues still require investigation. Most of the signaling pathways leading to actin reorganization that were described for T and B cells occur downstream to a single dominant receptor. NK signaling pathways are more complex due to the different constellations of activating or inhibiting receptor co-ligation, and the interplay between surface co-receptors that dictates NK cell stimulation. Much more study is needed to understand how NK cell signaling is tuned downstream to different receptor combinations (both activating and inhibitory), and how these influence cytoskeletal reorganization and receptor organization. In addition, we still know little regarding how different cytoskeletal regulators, namely NFs and NPFs play a role in shaping the architecture of the NKIS and the nano-scale organization of surface receptors. Genetic modulation of such factors in NK cell lines and primary NK cells may reveal their roles in coordinating the organization of receptors on the NK cell surface and influencing their activation states.

In recent years, it was shown that NK cells belong to a larger family of ILCs that have unique tissue distribution and function. NK cell heterogeneity is staggering, and it will be a future challenge to uncover how the cytoskeleton shapes the receptor organization and activity of distinct NK cell subtypes during homeostasis and pathology. Since many tissue-resident NK cells display a less cytotoxic phenotype, it is likely that their cytoskeleton is regulated differently and might arrange the architecture of surface receptors in a unique manner. Furthermore, it will be interesting to examine NK cytoskeletal dysfunction in diseases such as chronic viral infection.

Finally, most studies in immune cells have focused on how signaling proteins impact actin-based mechanisms, and the consequent effect of the actin meshwork on immune cell function. Only recently has the reciprocal integration and effect of the cytoskeletal machinery on signaling cascades begun to be investigated, specifically in the context of mechanotransduction. In NK cells, these issues remain even more enigmatic, yet it is clear that the robust and versatile nature of the actin cytoskeleton in NK cells plays additional roles beyond those described to date. Elucidating further functions of actin dynamics in NK cells may enhance our understanding of global NK cell signaling integration, and how this ultimately leads to NK cell development and function. Consequently, this raises the exciting possibility of actin modifying therapies for treating immune deficiencies and for immunotherapy.

## Author Contributions

AB-S, BS, and MB-S wrote the manuscript. All authors contributed to the article and approved the submitted version.

## Conflict of Interest

The authors declare that the research was conducted in the absence of any commercial or financial relationships that could be construed as a potential conflict of interest.

## References

[B1] Abdul-Manan N. Aghazadeh B. Liu G. A. Majumdar A. Ouerfelli O. Siminovitch K. A. (1999). Structure of Cdc42 in complex with the GTPase-binding domain of the ‘Wiskott-Aldrich syndrome’ protein. *Nature* 399 379–383. 10.1038/20726 10360578

[B2] Abel A. M. Tiwari A. A. Gerbec Z. J. Siebert J. R. Yang C. Schloemer N. J. (2018). IQ domain-containing GTPase-activating protein 1 regulates cytoskeletal reorganization and facilitates NKG2D-mediated mechanistic target of rapamycin complex 1 activation and cytokine gene translation in natural killer cells. *Front. Immunol.* 9:1168. 10.3389/fimmu.2018.01168 29892299 PMC5985319

[B3] Abeyweera T. P. Merino E. Huse M. (2011). Inhibitory signaling blocks activating receptor clustering and induces cytoskeletal retraction in natural killer cells. *J. Cell Biol.* 192 675–690. 10.1083/jcb.201009135 21339333 PMC3044118

[B4] Akhmanova A. Steinmetz M. O. (2015). Control of microtubule organization and dynamics: two ends in the limelight. *Nat. Rev. Mol. Cell Biol.* 16 711–726. 10.1038/nrm4084 26562752

[B5] Al Absi A. Wurzer H. Guerin C. Hoffmann C. Moreau F. Mao X. (2018). Actin cytoskeleton remodeling drives breast cancer cell escape from natural killer-mediated cytotoxicity. *Cancer Res.* 78 5631–5643. 10.1158/0008-5472.can-18-0441 30104240

[B6] Allavena P. (1991). Molecules and structures involved in the adhesion of natural killer cells to vascular endothelium. *J. Exp. Med.* 173 439–448. 10.1084/jem.173.2.439 1671081 PMC2118798

[B7] Andzelm M. M. Chen X. Krzewski K. Orange J. S. Strominger J. L. (2007). Myosin IIA is required for cytolytic granule exocytosis in human NK cells. *J. Exp. Med.* 204 2285–2291. 10.1084/jem.20071143 17875677 PMC2118468

[B8] Antoku S. Mayer B. J. (2009). Distinct roles for Crk adaptor isoforms in actin reorganization induced by extracellular signals. *J. Cell Sci.* 122 4228–4238. 10.1242/jcs.054627 19861495 PMC2776506

[B9] Azoulay-Alfaguter I. Strazza M. Peled M. Novak H. K. Muller J. Dustin M. L. (2017). The tyrosine phosphatase SHP-1 promotes T cell adhesion by activating the adaptor protein CrkII in the immunological synapse. *Sci. Signal.* 10:eaal2880. 10.1126/scisignal.aal2880 28790195 PMC9437928

[B10] Babich A. Li S. O’Connor R. S. Milone M. C. Freedman B. D. Burkhardt J. K. (2012). F-actin polymerization and retrograde flow drive sustained PLCγ1 signaling during T cell activation. *J. Cell Biol.* 197 775–787. 10.1083/jcb.201201018 22665519 PMC3373411

[B11] Bálint Š Lopes F. B. Davis D. M. (2018). A nanoscale reorganization of the IL-15 receptor is triggered by NKG2D in a ligand-dependent manner. *Sci. Signal.* 11:eaal3606. 10.1126/scisignal.aal3606 29636390 PMC6917509

[B12] Banerjee P. P. Orange J. S. (2010). Quantitative measurement of F-actin accumulation at the NK cell immunological synapse. *J. Immunol. Methods* 355 1–13. 10.1016/j.jim.2010.02.003 20171970 PMC2854315

[B13] Banerjee P. P. Pandey R. Zheng R. Suhoski M. M. Monaco-Shawver L. Orange J. S. (2007). Cdc42-interacting protein-4 functionally links actin and microtubule networks at the cytolytic NK cell immunological synapse. *J. Exp. Med.* 204 2305–2320. 10.1084/jem.20061893 17785506 PMC2118451

[B14] Barber D. F. Faure M. Long E. O. (2004). LFA-1 contributes an early signal for NK cell cytotoxicity. *J. Immunol.* 173 3653–3659. 10.4049/jimmunol.173.6.3653 15356110

[B15] Barda-Saad M. Braiman A. Titerence R. Bunnell S. C. Barr V. A. Samelson L. E. (2005). Dynamic molecular interactions linking the T cell antigen receptor to the actin cytoskeleton. *Nat. Immunol.* 6 80–89. 10.1038/ni1143 15558067

[B16] Barda-saad M. Shirasu N. Pauker M. H. Hassan N. Perl O. Balbo A. (2010). Cooperative interactions at the SLP-76 complex are critical for actin polymerization. *EMBO J.* 29 2315–2328. 10.1038/emboj.2010.133 20562827 PMC2910278

[B17] Barreira da Silva R. Graf C. Münz C. (2011). Cytoskeletal stabilization of inhibitory interactions in immunologic synapses of mature human dendritic cells with natural killer cells. *Blood* 118 6487–6498. 10.1182/blood-2011-07-366328 21917751 PMC3242715

[B18] Basu R. Whitlock B. M. Husson J. Le Floc’h A. Jin W. Oyler-Yaniv A. (2016). Cytotoxic T cells use mechanical force to potentiate target cell killing. *Cell* 165 100–110. 10.1016/j.cell.2016.01.021 26924577 PMC4808403

[B19] Ben-Shmuel A. Joseph N. Sabag B. Barda-Saad M. (2019). Lymphocyte mechanotransduction: the regulatory role of cytoskeletal dynamics in signaling cascades and effector functions. *J. Leukoc. Biol.* 105 1261–1273. 10.1002/JLB.MR0718-267R 30707462

[B20] Billadeau D. D. Brumbaugh K. M. Dick C. J. Schoon R. A. Bustelo X. R. Leibson P. J. (1998). The Vav-Rac1 pathway in cytotoxic lymphocytes regulates the generation of cell-mediated killing. *J. Exp. Med.* 188 549–559. 10.1084/jem.188.3.549 9687532 PMC2212464

[B21] Billadeau D. D. Upshaw J. L. Schoon R. A. Dick C. J. Leibson P. J. (2003). NKG2D-DAP10 triggers human NK cell-mediated killing via a Syk-independent regulatory pathway. *Nat. Immunol.* 4 557–564. 10.1038/ni929 12740575

[B22] Binstadt B. A. Billadeau D. D. Jevremovic D. Williams B. L. Fang N. Yi T. (1998). SLP-76 is a direct substrate of SHP-1 recruited to killer cell inhibitory receptors. *J. Biol. Chem.* 273 27518–27523. 10.1074/jbc.273.42.27518 9765283

[B23] Boztug K. Germeshausen M. Avedillo Díez I. Gulacsy V. Diestelhorst J. Ballmaier M. (2008). Multiple independent second-site mutations in two siblings with somatic mosaicism for Wiskott-Aldrich syndrome. *Clin. Genet.* 74 68–74. 10.1111/j.1399-0004.2008.01019.x 18479478

[B24] Brown A. C. N. Dobbie I. M. Alakoskela J.-M. Davis I. Davis D. M. (2012). Super-resolution imaging of remodeled synaptic actin reveals different synergies between NK cell receptors and integrins. *Blood* 120 3729–3740. 10.1182/blood-2012-05-429977 22966166 PMC4238744

[B25] Brown A. C. N. Oddos S. Dobbie I. M. Alakoskela J.-M. Parton R. M. Eissmann P. (2011). Remodelling of cortical actin where lytic granules dock at natural killer cell immune synapses revealed by super-resolution microscopy. *PLoS Biol.* 9:e1001152. 10.1371/journal.pbio.1001152 21931537 PMC3172219

[B26] Brugnera E. Haney L. Grimsley C. Lu M. Walk S. F. Tosello-Trampont A.-C. (2002). Unconventional Rac-GEF activity is mediated through the Dock180-ELMO complex. *Nat. Cell Biol.* 4 574–582. 10.1038/ncb824 12134158

[B27] Bryceson Y. T. March M. E. Ljunggren H.-G. Long E. O. (2006). Synergy among receptors on resting NK cells for the activation of natural cytotoxicity and cytokine secretion. *Blood* 107 159–166. 10.1182/blood-2005-04-1351 16150947 PMC1895346

[B28] Bryceson Y. T. Rudd E. Zheng C. Edner J. Ma D. Wood S. M. (2007). Defective cytotoxic lymphocyte degranulation in syntaxin-11-deficient familial hemophagocytic lymphohistiocytosis 4 (FHL4) patients. *Blood* 110 1906–1915. 10.1182/blood-2007-02-074468 17525286 PMC1976360

[B29] Burshtyn D. N. Shin J. Stebbins C. Long E. O. (2000). Adhesion to target cells is disrupted by the killer cell inhibitory receptor. *Curr. Biol.* 10 777–780. 10.1016/s0960-9822(00)00568-6 10898979

[B30] Butler B. Cooper J. A. (2009). Distinct roles for the actin nucleators Arp2/3 and hDia1 during NK-mediated cytotoxicity. *Curr. Biol.* 19 1886–1896. 10.1016/j.cub.2009.10.029 19913427 PMC2937835

[B31] Butler B. Kastendieck D. H. Cooper J. A. (2008). Differently phosphorylated forms of the cortactin homolog HS1 mediate distinct functions in natural killer cells. *Nat. Immunol.* 9 887–897. 10.1038/ni.1630 18587398 PMC2622733

[B32] Carisey A. F. Mace E. M. Saeed M. B. Davis D. M. Orange J. S. (2018). Nanoscale dynamism of actin enables secretory function in cytolytic cells. *Curr. Biol.* 28 489–502.e9. 29398219 10.1016/j.cub.2017.12.044 PMC5835143

[B33] Carpén O. Virtanen I. Lehto V. P. Saksela E. (1983). Polarization of NK cell cytoskeleton upon conjugation with sensitive target cells. *J. Immunol.* 131 2695–2698. 6417230

[B34] Catucci M. Zanoni I. Draghici E. Bosticardo M. Castiello M. C. Venturini M. (2014). Wiskott-Aldrich syndrome protein deficiency in natural killer and dendritic cells affects antitumor immunity. *Eur. J. Immunol.* 44 1039–1045. 10.1002/eji.201343935 24338698

[B35] Cella M. Fujikawa K. Tassi I. Kim S. Latinis K. Nishi S. (2004). Differential requirements for Vav proteins in DAP10- and ITAM-mediated NK cell cytotoxicity. *J. Exp. Med.* 200 817–823. 10.1084/jem.20031847 15365099 PMC2211968

[B36] Chen R. Relouzat F. Roncagalli R. Aoukaty A. Tan R. Latour S. (2004). Molecular dissection of 2B4 signaling: implications for signal transduction by SLAM-related receptors. *Mol. Cell. Biol.* 24 5144–5156. 10.1128/mcb.24.12.5144-5156.2004 15169881 PMC419855

[B37] Chen X. Allan D. S. J. Krzewski K. Ge B. Kopcow H. Strominger J. L. (2006). CD28-stimulated ERK2 phosphorylation is required for polarization of the microtubule organizing center and granules in YTS NK cells. *Proc. Natl. Acad. Sci. U.S.A.* 103 10346–10351. 10.1073/pnas.0604236103 16801532 PMC1502460

[B38] Chen X. Trivedi P. P. Ge B. Krzewski K. Strominger J. L. (2007). Many NK cell receptors activate ERK2 and JNK1 to trigger microtubule organizing center and granule polarization and cytotoxicity. *Proc. Natl. Acad. Sci. U.S.A.* 104 6329–6334. 10.1073/pnas.0611655104 17395718 PMC1851054

[B39] Chereau D. Kerff F. Graceffa P. Grabarek Z. Langsetmo K. Dominguez R. (2005). Actin-bound structures of Wiskott-Aldrich syndrome protein (WASP)-homology domain 2 and the implications for filament assembly. *Proc. Natl. Acad. Sci. U.S.A.* 102 16644–16649. 10.1073/pnas.0507021102 16275905 PMC1283820

[B40] Choudhuri K. Llodrá J. Roth E. W. Tsai J. Gordo S. Wucherpfennig K. W. (2014). Polarized release of T-cell-receptor-enriched microvesicles at the immunological synapse. *Nature* 507 118–123. 10.1038/nature12951 24487619 PMC3949170

[B41] Comrie W. A. Babich A. Burkhardt J. K. (2015a). F-actin flow drives affinity maturation and spatial organization of LFA-1 at the immunological synapse. *J. Cell Biol.* 208 475–491. 10.1083/jcb.201406121 25666810 PMC4332248

[B42] Comrie W. A. Li S. Boyle S. Burkhardt J. K. (2015b). The dendritic cell cytoskeleton promotes T cell adhesion and activation by constraining ICAM-1 mobility. *J. Cell Biol.* 208 457–473. 10.1083/jcb.201406120 25666808 PMC4332244

[B43] Comrie W. A. Burkhardt J. K. (2016). Action and traction: cytoskeletal control of receptor triggering at the immunological synapse. *Front. Immunol.* 7:68. 10.3389/fimmu.2016.00068 27014258 PMC4779853

[B44] Cory G. O. C. Garg R. Cramer R. Ridley A. J. (2002). Phosphorylation of tyrosine 291 enhances the ability of WASp to stimulate actin polymerization and filopodium formation. Wiskott-aldrich syndrome protein. *J. Biol. Chem.* 277 45115–45121. 10.1074/jbc.m203346200 12235133

[B45] Côté J.-F. Vuori K. (2002). Identification of an evolutionarily conserved superfamily of DOCK180-related proteins with guanine nucleotide exchange activity. *J. Cell Sci.* 115 (Pt 24) 4901–4913. 10.1242/jcs.00219 12432077

[B46] Côte M. Ménager M. M. Burgess A. Mahlaoui N. Picard C. Schaffner C. (2009). Munc18-2 deficiency causes familial hemophagocytic lymphohistiocytosis type 5 and impairs cytotoxic granule exocytosis in patient NK cells. *J. Clin. Invest.* 119 3765–3773. 10.1172/jci40732 19884660 PMC2786810

[B47] Culley F. J. Johnson M. Evans J. H. Kumar S. Crilly R. Casasbuenas J. (2009). Natural killer cell signal integration balances synapse symmetry and migration. *PLoS Biol.* 7:e1000159. 10.1371/journal.pbio.1000159 19636352 PMC2707003

[B48] Davis D. M. (2009). Mechanisms and functions for the duration of intercellular contacts made by lymphocytes. *Nat. Rev. Immunol.* 9 543–555. 10.1038/nri2602 19609264

[B49] Davis D. M. Chiu I. Fassett M. Cohen G. B. Mandelboim O. Strominger J. L. (1999). The human natural killer cell immune synapse. *Proc. Natl. Acad. Sci. U.S.A.* 96 15062–15067. 10611338 10.1073/pnas.96.26.15062 PMC24773

[B50] de la Fuente M. A. Sasahara Y. Calamito M. Antón I. M. Elkhal A. Gallego M. D. (2007). WIP is a chaperone for Wiskott-Aldrich syndrome protein (WASP). *Proc. Natl. Acad. Sci. U.S.A.* 104 926–931. 17213309 10.1073/pnas.0610275104 PMC1783416

[B51] Delcassian D. Depoil D. Rudnicka D. Liu M. Davis D. M. Dustin M. L. (2013). Nanoscale ligand spacing influences receptor triggering in T cells and NK cells. *Nano Lett.* 13 5608–5614. 10.1021/nl403252x 24125583 PMC4288448

[B52] Dell’Angelica E. C. Ohno H. Ooi C. E. Rabinovich E. Roche K. W. Bonifacino J. S. (1997). AP-3: an adaptor-like protein complex with ubiquitous expression. *EMBO J.* 16 917–928. 10.1093/emboj/16.5.917 9118953 PMC1169692

[B53] Dell’Angelica E. C. Shotelersuk V. Aguilar R. C. Gahl W. A. Bonifacino J. S. (1999). Altered trafficking of lysosomal proteins in hermansky-pudlak syndrome due to mutations in the β3A subunit of the AP-3 adaptor. *Mol. Cell* 3 11–21. 10.1016/s1097-2765(00)80170-7 10024875

[B54] Derry J. M. J. Ochs H. D. Francke U. (1994). Isolation of a novel gene mutated in Wiskott-Aldrich syndrome. *Cell* 78 635–644. 10.1016/0092-8674(94)90528-2 8069912

[B55] Desai A. Verma S. Mitchison T. J. Walczak C. E. (1999). Kin I kinesins are microtubule-destabilizing enzymes. *Cell* 96 69–78. 10.1016/s0092-8674(00)80960-5 9989498

[B56] Dobbs K. Domínguez Conde C. Zhang S.-Y. Parolini S. Audry M. Chou J. (2015). Inherited DOCK2 deficiency in patients with early-onset invasive infections. *N. Engl. J. Med.* 372 2409–2422. 26083206 10.1056/NEJMoa1413462 PMC4480434

[B57] Dong Z. Davidson D. Pérez-Quintero L. A. Kurosaki T. Swat W. Veillette A. (2012). The adaptor SAP controls NK cell activation by regulating the enzymes Vav-1 and SHIP-1 and by enhancing conjugates with target cells. *Immunity* 36 974–985. 10.1016/j.immuni.2012.03.023 22683124

[B58] Downward J. Graves J. D. Warne P. H. Rayter S. Cantrell D. A. (1990). Stimulation of p21ras upon T-cell activation. *Nature* 346 719–723. 10.1038/346719a0 2201921

[B59] Duan X. Lu J. Wang H. Liu X. Wang J. Zhou K. (2017). Bidirectional factors impact the migration of NK cells to draining lymph node in aged mice during influenza virus infection. *Exp. Gerontol.* 96 127–137. 10.1016/j.exger.2017.06.021 28669820

[B60] Dulyaninova N. G. Malashkevich V. N. Almo S. C. Bresnick A. R. (2005). Regulation of Myosin-IIA assembly and Mts1 binding by heavy chain phosphorylation ^†^. *Biochemistry* 44 6867–6876. 10.1021/bi0500776 15865432

[B61] Dustin M. L. (2014). The immunological synapse. *Cancer Immunol. Res.* 2 1023–1033. 25367977 10.1158/2326-6066.CIR-14-0161 PMC4692051

[B62] Dustin M. L. Chakraborty A. K. Shaw A. S. (2010). Understanding the structure and function of the immunological synapse. *Cold Spring Harb. Perspect. Biol.* 2:a002311. 10.1101/cshperspect.a002311 20843980 PMC2944359

[B63] Dustin M. L. Long E. O. (2010). Cytotoxic immunological synapses. *Immunol. Rev.* 235 24–34. 10.1111/j.0105-2896.2010.00904.x 20536553 PMC2950621

[B64] Eisenmann K. M. West R. A. Hildebrand D. Kitchen S. M. Peng J. Sigler R. (2007). T cell responses in mammalian diaphanous-related formin mDia1 knock-out mice. *J. Biol. Chem.* 282 25152–25158. 10.1074/jbc.m703243200 17595162

[B65] Eissmann P. Beauchamp L. Wooters J. Tilton J. C. Long E. O. Watzl C. (2005). Molecular basis for positive and negative signaling by the natural killer cell receptor 2B4 (CD244). *Blood* 105 4722–4729. 10.1182/blood-2004-09-3796 15713798

[B66] Eissmann P. Watzl C. (2006). Molecular analysis of NTB-A signaling: a role for EAT-2 in NTB-A-mediated activation of human NK cells. *J. Immunol.* 177 3170–3177. 10.4049/jimmunol.177.5.3170 16920955

[B67] El-Shazly A. E. Doloriert H. C. Bisig B. Lefebvre P. P. Delvenne P. Jacobs N. (2013). Novel cooperation between CX3CL1 and CCL26 inducing NK cell chemotaxis via CX3CR1: a possible mechanism for NK cell infiltration of the allergic nasal tissue. *Clin. Exp. Allergy* 43 322–331. 10.1111/cea.12022 23414540

[B68] Elstak E. D. Neeft M. Nehme N. T. Voortman J. Cheung M. Goodarzifard M. (2011). The munc13-4-rab27 complex is specifically required for tethering secretory lysosomes at the plasma membrane. *Blood* 118 1570–1578. 10.1182/blood-2011-02-339523 21693760

[B69] Enqvist M. Ask E. H. Forslund E. Carlsten M. Abrahamsen G. Beziat V. (2015). Coordinated expression of DNAM-1 and LFA-1 in educated NK cells. *J. Immunol.* 194 4518–4527. 10.4049/jimmunol.1401972 25825444

[B70] Fassett M. S. Davis D. M. Valter M. M. Cohen G. B. Strominger J. L. (2001). Signaling at the inhibitory natural killer cell immune synapse regulates lipid raft polarization but not class I MHC clustering. *Proc. Natl. Acad. Sci. U.S.A.* 98 14547–14552. 10.1073/pnas.211563598 11724921 PMC64719

[B71] Faure M. Barber D. F. Takahashi S. M. Jin T. Long E. O. (2003). Spontaneous clustering and tyrosine phosphorylation of NK cell inhibitory receptor induced by ligand binding. *J. Immunol.* 170 6107–6114. 10.4049/jimmunol.170.12.6107 12794140

[B72] Feldmann J. Callebaut I. Raposo G. Certain S. Bacq D. Dumont C. (2003). Munc13-4 is essential for cytolytic granules fusion and is mutated in a form of familial hemophagocytic lymphohistiocytosis (FHL3). *Cell* 115 461–473. 10.1016/s0092-8674(03)00855-9 14622600

[B73] Fogler W. E. Volker K. McCormick K. L. Watanabe M. Ortaldo J. R. Wiltrout R. H. (1996). NK cell infiltration into lung, liver, and subcutaneous B16 melanoma is mediated by VCAM-1/VLA-4 interaction. *J. Immunol.* 156 4707–4714. 8648116

[B74] Fontana S. Parolini S. Vermi W. Booth S. Gallo F. Donini M. (2006). Innate immunity defects in hermansky-pudlak type 2 syndrome. *Blood* 107 4857–4864. 10.1182/blood-2005-11-4398 16507770

[B75] Fried S. Matalon O. Noy E. Barda-Saad M. (2014a). WIP: more than a WASp-interacting protein. *J. Leukoc. Biol.* 96 713–727. 10.1189/jlb.2ru0314-162r 25210148

[B76] Fried S. Reicher B. Pauker M. H. Eliyahu S. Matalon O. Noy E. (2014b). Triple-color FRET analysis reveals conformational changes in the WIP-WASp actin-regulating complex. *Sci. Signal.* 7:ra60. 10.1126/scisignal.2005198 24962707

[B77] Galjart N. (2005). CLIPs and CLASPs and cellular dynamics. *Nat. Rev. Mol. Cell Biol.* 6 487–498. 10.1038/nrm1664 15928712

[B78] Gardner M. K. Zanic M. Gell C. Bormuth V. Howard J. (2011). Depolymerizing kinesins Kip3 and MCAK shape cellular microtubule architecture by differential control of catastrophe. *Cell* 147 1092–1103. 10.1016/j.cell.2011.10.037 22118464

[B79] Garrod K. R. Wei S. H. Parker I. Cahalan M. D. (2007). Natural killer cells actively patrol peripheral lymph nodes forming stable conjugates to eliminate MHC-mismatched targets. *Proc. Natl. Acad. Sci. U.S.A.* 104 12081–12086. 10.1073/pnas.0702867104 17609379 PMC1924574

[B80] Gasman S. Kalaidzidis Y. Zerial M. (2003). RhoD regulates endosome dynamics through Diaphanous-related Formin and Src tyrosine kinase. *Nat. Cell Biol.* 5 195–204. 10.1038/ncb935 12577064

[B81] Gil-Krzewska A. Saeed M. B. Oszmiana A. Fischer E. R. Lagrue K. Gahl W. A. (2017). An actin cytoskeletal barrier inhibits lytic granule release from natural killer cells in patients with Chediak-Higashi syndrome. *J. Allergy Clin. Immunol.* 142 914–927.e6. 10.1016/j.jaci.2017.10.040 29241728 PMC5995607

[B82] Gismondi A. Bisogno L. Mainiero F. Palmieri G. Piccoli M. Frati L. (1997). Proline-rich tyrosine kinase-2 activation by beta 1 integrin fibronectin receptor cross-linking and association with paxillin in human natural killer cells. *J. Immunol.* 159 4729–4736. 9366396

[B83] Gismondi A. Cifaldi L. Mazza C. Giliani S. Parolini S. Morrone S. (2004). Impaired natural and CD16-mediated NK cell cytotoxicity in patients with WAS and XLT: ability of IL-2 to correct NK cell functional defect. *Blood* 104 436–443. 10.1182/blood-2003-07-2621 15001467

[B84] Giurisato E. Cella M. Takai T. Kurosaki T. Feng Y. Longmore G. D. (2007). Phosphatidylinositol 3-kinase activation is required to form the NKG2D immunological synapse. *Mol. Cell. Biol.* 27 8583–8599. 10.1128/mcb.01477-07 17923698 PMC2169389

[B85] Gomez T. S. Kumar K. Medeiros R. B. Shimizu Y. Leibson P. J. Billadeau D. D. (2007). Formins regulate the actin-related protein 2/3 complex-independent polarization of the centrosome to the immunological synapse. *Immunity* 26 177–190. 10.1016/j.immuni.2007.01.008 17306570 PMC2836258

[B86] Graham D. B. Cella M. Giurisato E. Fujikawa K. Miletic A. V. Kloeppel T. (2006). Vav1 Controls DAP10-mediated natural cytotoxicity by regulating actin and microtubule dynamics. *J. Immunol.* 177 2349–2355. 10.4049/jimmunol.177.4.2349 16887996

[B87] Gross C. C. Brzostowski J. A. Liu D. Long E. O. (2010). Tethering of intercellular adhesion molecule on target cells is required for LFA-1-dependent NK cell adhesion and granule polarization. *J. Immunol.* 185 2918–2926. 10.4049/jimmunol.1000761 20675589 PMC3867939

[B88] Guia S. Jaeger B. N. Piatek S. Mailfert S. Trombik T. Fenis A. (2011). Confinement of activating receptors at the plasma membrane controls natural killer cell tolerance. *Sci. Signal.* 4:ra21. 10.1126/scisignal.2001608 21467299

[B89] Hadad U. Thauland T. J. Martinez O. M. Butte M. J. Porgador A. Krams S. M. (2015). NKp46 clusters at the immune synapse and regulates NK cell polarization. *Front. Immunol.* 6:495. 10.3389/fimmu.2015.00495 26441997 PMC4585260

[B90] Ham H. Billadeau D. D. (2014). Human immunodeficiency syndromes affecting human natural killer cell cytolytic activity. *Front. Immunol.* 5:2. 10.3389/fimmu.2014.00002 24478771 PMC3896857

[B91] Ham H. Guerrier S. Kim J. Schoon R. A. Anderson E. L. Hamann M. J. (2013). Dedicator of cytokinesis 8 interacts with talin and Wiskott-Aldrich syndrome protein to regulate NK cell cytotoxicity. *J. Immunol.* 190 3661–3669. 10.4049/jimmunol.1202792 23455509 PMC3841075

[B92] Ham H. Huynh W. Schoon R. A. Vale R. D. Billadeau D. D. (2015). HkRP3 is a microtubule-binding protein regulating lytic granule clustering and NK cell killing. *J. Immunol.* 194 3984–3996. 10.4049/jimmunol.1402897 25762780 PMC4390494

[B93] Hammer J. A. Wang J. Saeed M. Pedrosa A. (2019). Origin, organization, dynamics, and function of Actin and Actomyosin networks at the T cell immunological synapse. *Annu. Rev. Immunol.* 37 201–224. 10.1146/annurev-immunol-042718-041341 30576253 PMC8343269

[B94] Han J. (1998). Role of substrates and products of PI 3-kinase in regulating activation of rac-related guanosine Triphosphatases by Vav. *Science* 279 558–560. 10.1126/science.279.5350.558 9438848

[B95] Harada Y. Tanaka Y. Terasawa M. Pieczyk M. Habiro K. Katakai T. (2012). DOCK8 is a Cdc42 activator critical for interstitial dendritic cell migration during immune responses. *Blood* 119 4451–4461. 10.1182/blood-2012-01-407098 22461490 PMC3418773

[B96] Hashimoto-tane A. Yokosuka T. Sakata-sogawa K. Sakuma M. Ishihara C. (2011). Article dynein-driven transport of T cell receptor microclusters regulates immune synapse formation and T cell activation. *Immunity* 34 919–931. 10.1016/j.immuni.2011.05.012 21703543

[B97] Hendricks A. G. Lazarus J. E. Perlson E. Gardner M. K. Odde D. J. Goldman Y. E. (2012). Dynein tethers and stabilizes dynamic microtubule plus ends. *Curr. Biol.* 22 632–637. 10.1016/j.cub.2012.02.023 22445300 PMC3347920

[B98] Hoffmann S. C. Cohnen A. Ludwig T. Watzl C. (2011). 2B4 engagement mediates rapid LFA-1 and actin-dependent NK cell adhesion to tumor cells as measured by single cell force spectroscopy. *J. Immunol.* 186 2757–2764. 10.4049/jimmunol.1002867 21270395

[B99] Hsu H.-T. Mace E. M. Carisey A. F. Viswanath D. I. Christakou A. E. Wiklund M. (2016). NK cells converge lytic granules to promote cytotoxicity and prevent bystander killing. *J. Cell Biol.* 215 875–889. 10.1083/jcb.201604136 27903610 PMC5166499

[B100] Hsu K. C. Keever-Taylor C. A. Wilton A. Pinto C. Heller G. Arkun K. (2005). Improved outcome in HLA-identical sibling hematopoietic stem-cell transplantation for acute myelogenous leukemia predicted by KIR and HLA genotypes. *Blood* 105 4878–4884. 10.1182/blood-2004-12-4825 15731175 PMC1894998

[B101] Huang L. Zhu P. Xia P. Fan Z. (2016). WASH has a critical role in NK cell cytotoxicity through Lck-mediated phosphorylation. *Cell Death Dis.* 7:e2301. 10.1038/cddis.2016.212 27441653 PMC4973352

[B102] Huang W. Ochs H. D. Dupont B. Vyas Y. M. (2005). The Wiskott-Aldrich syndrome protein regulates nuclear translocation of NFAT2 and NF-kappa B (RelA) independently of its role in filamentous actin polymerization and actin cytoskeletal rearrangement. *J. Immunol.* 174 2602–2611. 10.4049/jimmunol.174.5.2602 15728466

[B103] Humphries C. L. Balcer H. I. D’Agostino J. L. Winsor B. Drubin D. G. Barnes G. (2002). Direct regulation of Arp2/3 complex activity and function by the actin binding protein coronin. *J. Cell Biol.* 159 993–1004. 10.1083/jcb.200206113 12499356 PMC2173993

[B104] Huse M. (2017). Mechanical forces in the immune system. *Nat. Rev. Immunol.* 17 679–690. 10.1038/nri.2017.74 28757604 PMC6312705

[B105] Hammer J. A. Burkhardt J. K. (2013). Controversy and consensus regarding myosin II function at the immunological synapse. *Curr. Opin. Immunol.* 25 300–306. 10.1016/j.coi.2013.03.010 23623641 PMC3691351

[B106] Iizuka Y. Cichocki F. Sieben A. Sforza F. Karim R. Coughlin K. (2015). UNC-45A Is a nonmuscle myosin IIA chaperone required for NK Cell cytotoxicity via control of lytic granule secretion. *J. Immunol.* 195 4760–4770. 10.4049/jimmunol.1500979 26438524 PMC5189640

[B107] Imai K. Matsuyama S. Miyake S. Suga K. Nakachi K. (2000). Natural cytotoxic activity of peripheral-blood lymphocytes and cancer incidence: an 11-year follow-up study of a general population. *Lancet* 356 1795–1799. 10.1016/s0140-6736(00)03231-1 11117911

[B108] Imai K. Morio T. Zhu Y. Jin Y. Itoh S. Kajiwara M. (2004). Clinical course of patients with WASP gene mutations. *Blood* 103 456–464. 10.1182/blood-2003-05-1480 12969986

[B109] Imai K. Nonoyama S. Ochs H. D. (2003). WASP (Wiskott-Aldrich syndrome protein) gene mutations and phenotype. *Curr. Opin. Allergy Clin. Immunol.* 3 427–436. 10.1097/00130832-200312000-00003 14612666

[B110] Introne W. Boissy R. E. Gahl W. A. (1999). Clinical, molecular, and cell biological aspects of chediak-higashi syndrome. *Mol. Genet. Metab.* 68 283–303. 10.1006/mgme.1999.2927 10527680

[B111] Ishigami S. Natsugoe S. Tokuda K. Nakajo A. Che X. Iwashige H. (2000). Prognostic value of intratumoral natural killer cells in gastric carcinoma. *Cancer* 88 577–583. 10.1002/(sici)1097-0142(20000201)88:3<577::aid-cncr13>3.0.co;2-v 10649250

[B112] Jacobelli J. Chmura S. A. Buxton D. B. Davis M. M. Krummel M. F. (2004). A single class II myosin modulates T cell motility and stopping, but not synapse formation. *Nat. Immunol.* 5 531–538. 10.1038/ni1065 15064761

[B113] James A. M. Hsu H.-T. Dongre P. Uzel G. Mace E. M. Banerjee P. P. (2013). Rapid activation receptor- or IL-2-induced lytic granule convergence in human natural killer cells requires Src, but not downstream signaling. *Blood* 121 2627–2637. 10.1182/blood-2012-06-437012 23380740 PMC3617630

[B114] Jankowska K. I. Williamson E. K. Roy N. H. Blumenthal D. Chandra V. Baumgart T. (2018). Integrins modulate T cell receptor signaling by constraining actin flow at the immunological synapse. *Front. Immunol.* 9:25. 10.3389/fimmu.2018.00025 29403502 PMC5778112

[B115] Jevremovic D. Billadeau D. D. Schoon R. A. Dick C. J. Irvin B. J. Zhang W. (1999). Cutting edge: a role for the adaptor protein LAT in human NK Cell-mediated cytotoxicity. *J. Immunol.* 162 2453–2456. 10072481

[B116] Johnson K. G. Bromley S. K. Dustin M. L. Thomas M. L. (2000). A supramolecular basis for CD45 tyrosine phosphatase regulation in sustained T cell activation. *Proc. Natl. Acad. Sci. U.S.A.* 97 10138–10143. 10.1073/pnas.97.18.10138 10963676 PMC27752

[B117] Kanwar N. Wilkins J. A. (2011). IQGAP1 involvement in MTOC and granule polarization in NK-cell cytotoxicity. *Eur. J. Immunol.* 41 2763–2773. 10.1002/eji.201040444 21681737

[B118] Karim M. A. Suzuki K. Fukai K. Oh J. Nagle D. L. Moore K. J. (2002). Apparent genotype-phenotype correlation in childhood, adolescent, and adult Chediak-Higashi syndrome. *Am. J. Med. Genet.* 108 16–22. 10.1002/ajmg.10184 11857544

[B119] Katz P. Zaytoun A. M. Lee J. H. (1982). Mechanisms of human cell-mediated cytotoxicity. III. Dependence of natural killing on microtubule and microfilament integrity. *J. Immunol.* 129 2816–2825. 6890568

[B120] Kennedy P. R. Barthen C. Williamson D. J. Pitkeathly W. T. E. Hazime K. S. Cumming J. (2019). Genetic diversity affects the nanoscale membrane organization and signaling of natural killer cell receptors. *Sci. Signal.* 12:eaaw9252. 10.1126/scisignal.aaw9252 31848320 PMC6944503

[B121] Kim A. S. Kakalis L. T. Abdul-Manan N. Liu G. A. Rosen M. K. (2000). Autoinhibition and activation mechanisms of the Wiskott-Aldrich syndrome protein. *Nature* 404 151–158. 10.1038/35004513 10724160

[B122] Kim H. S. Das A. Gross C. C. Bryceson Y. T. Long E. O. (2010). Synergistic signals for natural cytotoxicity are required to overcome inhibition by c-Cbl ubiquitin ligase. *Immunity* 32 175–186. 10.1016/j.immuni.2010.02.004 20189481 PMC2843589

[B123] Kim H. S. Long E. O. (2012). Complementary phosphorylation sites in the adaptor protein SLP-76 promote synergistic activation of natural killer cells. *Sci. Signal.* 5:ra49. 10.1126/scisignal.2002754 22786724 PMC3842037

[B124] Kloc M. Kubiak J. Z. Li X. C. Ghobrial R. M. (2014). The newly found functions of MTOC in immunological response. *J. Leukoc. Biol.* 95 417–430. 10.1189/jlb.0813468 24295827

[B125] Kopcow H. D. Allan D. S. J. Chen X. Rybalov B. Andzelm M. M. Ge B. (2005). Human decidual NK cells form immature activating synapses and are not cytotoxic. *Proc. Natl. Acad. Sci. U.S.A.* 102 15563–15568. 10.1073/pnas.0507835102 16230631 PMC1266146

[B126] Kritikou J. S. Dahlberg C. I. M. Baptista M. A. P. Wagner A. K. Banerjee P. P. Gwalani L. A. (2016). IL-2 in the tumor microenvironment is necessary for Wiskott-Aldrich syndrome protein deficient NK cells to respond to tumors in vivo. *Sci. Rep.* 6:30636. 10.1038/srep30636 PMC4967920 27477778

[B127] Krzewski K. Chen X. Orange J. S. Strominger J. L. (2006). Formation of a WIP-, WASp-, actin-, and myosin IIA-containing multiprotein complex in activated NK cells and its alteration by KIR inhibitory signaling. *J. Cell Biol.* 173 121–132. 10.1083/jcb.200509076 16606694 PMC2063796

[B128] Krzewski K. Chen X. Strominger J. L. (2008). WIP is essential for lytic granule polarization and NK cell cytotoxicity. *Proc. Natl. Acad. Sci. U.S.A.* 105 2568–2573. 10.1073/pnas.0711593105 18258743 PMC2268177

[B129] Kueh H. Y. Charras G. T. Mitchison T. J. Brieher W. M. (2008). Actin disassembly by cofilin, coronin, and Aip1 occurs in bursts and is inhibited by barbed-end cappers. *J. Cell Biol.* 182 341–353. 10.1083/jcb.200801027 18663144 PMC2483518

[B130] Kumari S. Depoil D. Martinelli R. Judokusumo E. Carmona G. Gertler F. B. (2015). Actin foci facilitate activation of the phospholipase C-γ in primary T lymphocytes via the WASP pathway. *eLife* 4:e04953. 10.7554/eLife.04953 PMC4355629 25758716

[B131] Kumari S. Vardhana S. Cammer M. Curado S. Santos L. Sheetz M. P. (2012). T Lymphocyte Myosin IIA is required for maturation of the immunological synapse. *Front. Immunol.* 3:230. 10.3389/fimmu.2012.00230 22912631 PMC3421155

[B132] Kurowska M. Goudin N. Nehme N. T. Court M. Garin J. Fischer A. (2012). Terminal transport of lytic granules to the immune synapse is mediated by the kinesin-1/Slp3/Rab27a complex. *Blood* 119 3879–3889. 10.1182/blood-2011-09-382556 22308290 PMC3824283

[B133] Lagrue K. Carisey A. Morgan D. J. Chopra R. Davis D. M. (2015). Lenalidomide augments actin remodeling and lowers NK-cell activation thresholds. *Blood* 126 50–60. 10.1182/blood-2015-01-625004 26002964 PMC4551357

[B134] Lagrue K. Carisey A. Oszmiana A. Kennedy P. R. Williamson D. J. Cartwright A. (2013). The central role of the cytoskeleton in mechanisms and functions of the NK cell immune synapse. *Immunol. Rev.* 256 203–221. 10.1111/imr.12107 24117823

[B135] Lämmermann T. Bader B. L. Monkley S. J. Worbs T. Wedlich-Söldner R. Hirsch K. (2008). Rapid leukocyte migration by integrin-independent flowing and squeezing. *Nature* 453 51–55. 10.1038/nature06887 18451854

[B136] Lanier L. L. (1998). NK cell receptors. *Annu. Rev. Immunol.* 16 359–393. 9597134 10.1146/annurev.immunol.16.1.359

[B137] Lanier L. L. (2003). Natural killer cell receptor signaling. *Curr. Opin. Immunol.* 15 308–314. 10.1016/s0952-7915(03)00039-6 12787756

[B138] Lanzi G. Moratto D. Vairo D. Masneri S. Delmonte O. Paganini T. (2012). A novel primary human immunodeficiency due to deficiency in the WASP-interacting protein WIP. *J. Exp. Med.* 209 29–34. 10.1084/jem.20110896 22231303 PMC3260865

[B139] Latour S. Roncagalli R. Chen R. Bakinowski M. Shi X. Schwartzberg P. L. (2003). Binding of SAP SH2 domain to FynT SH3 domain reveals a novel mechanism of receptor signalling in immune regulation. *Nat. Cell Biol.* 5 149–154. 10.1038/ncb919 12545173

[B140] Lee S.-H. Miyagi T. Biron C. A. (2007). Keeping NK cells in highly regulated antiviral warfare. *Trends Immunol.* 28 252–259. 10.1016/j.it.2007.04.001 17466596

[B141] Liu D. Meckel T. Long E. O. (2010). Distinct role of Rab27a in granule movement at the plasma membrane and in the cytosol of NK cells. *PLoS One* 5:e12870 10.1371/journal.pbio.10012870 PMC2943471 20877725

[B142] Liu D. Peterson M. E. Long E. O. (2012). The adaptor protein Crk controls activation and inhibition of natural killer cells. *Immunity* 36 600–611. 10.1016/j.immuni.2012.03.007 22464172 PMC3355982

[B143] Long E. O. (1999). Regulation of immune responses through inhibitory receptors. *Annu. Rev. Immunol.* 17 875–904. 10.1146/annurev.immunol.17.1.875 10358776

[B144] Long E. O. (2008). Negative signaling by inhibitory receptors: the NK cell paradigm. *Immunol. Rev.* 224 70–84. 10.1111/j.1600-065x.2008.00660.x 18759921 PMC2587243

[B145] Long E. O. Kim H. S. Liu D. Peterson M. E. Rajagopalan S. (2013). Controlling natural killer cell responses: integration of signals for activation and inhibition. *Annu. Rev. Immunol.* 31 227–258. 10.1146/annurev-immunol-020711-075005 23516982 PMC3868343

[B146] Lozano M. L. Rivera J. Sánchez-Guiu I. Vicente V. (2014). Towards the targeted management of chediak-higashi syndrome. *Orphanet J. Rare Dis.* 9:132. 10.1186/s13023-014-0132-6 PMC4243965 25129365

[B147] Mace E. M. Orange J. S. (2014). Lytic immune synapse function requires filamentous actin deconstruction by Coronin 1A. *Proc. Natl. Acad. Sci. U.S.A.* 111 6708–6713. 10.1073/pnas.1314975111 24760828 PMC4020046

[B148] Mace E. M. Wu W. W. Ho T. Mann S. S. Hsu H.-T. Orange J. S. (2012). NK cell lytic granules are highly motile at the immunological synapse and require F-actin for post-degranulation persistence. *J. Immunol.* 189 4870–4880. 10.4049/jimmunol.1201296 23066148 PMC3558996

[B149] Mace E. M. Zhang J. Siminovitch K. A. Takei F. (2010). Elucidation of the integrin LFA-1-mediated signaling pathway of actin polarization in natural killer cells. *Blood* 116 1272–1279. 10.1182/blood-2009-12-261487 20472831

[B150] Maravillas-Montero J. L. Santos-Argumedo L. (2012). The myosin family: unconventional roles of actin-dependent molecular motors in immune cells. *J. Leukoc. Biol.* 91 35–46. 10.1189/jlb.0711335 21965174

[B151] Marcenaro S. Gallo F. Martini S. Santoro A. Griffiths G. M. Aricó M. (2006). Analysis of natural killer-cell function in familial hemophagocytic lymphohistiocytosis (FHL): defective CD107a surface expression heralds Munc13-4 defect and discriminates between genetic subtypes of the disease. *Blood* 108 2316–2323. 10.1182/blood-2006-04-015693 16778144

[B152] March M. E. Long E. O. (2011). 2 Integrin induces TCR -Syk-Phospholipase C- phosphorylation and paxillin-dependent granule polarization in human NK cells. *J. Immunol.* 186 2998–3005. 10.4049/jimmunol.1002438 21270398 PMC3845901

[B153] Martín-Cófreces N. B. Baixauli F. López M. J. Gil D. Monjas A. Alarcón B. (2012). End-binding protein 1 controls signal propagation from the T cell receptor. *EMBO J.* 31 4140–4152. 10.1038/emboj.2012.242 22922463 PMC3492726

[B154] Martín-Cófreces N. B. Robles-Valero J. Cabrero J. R. Mittelbrunn M. Gordón-Alonso M. Sung C.-H. (2008). MTOC translocation modulates IS formation and controls sustained T cell signaling. *J. Cell Biol.* 182 951–962. 10.1083/jcb.200801014 18779373 PMC2528574

[B155] Martín-Fontecha A. Thomsen L. L. Brett S. Gerard C. Lipp M. Lanzavecchia A. (2004). Induced recruitment of NK cells to lymph nodes provides IFN-gamma for T(H)1 priming. *Nat. Immunol.* 5 1260–1265. 10.1038/ni1138 15531883

[B156] Masilamani M. Nguyen C. Kabat J. Borrego F. Coligan J. E. (2006). CD94/NKG2A inhibits NK cell activation by disrupting the actin network at the immunological synapse. *J. Immunol.* 177 3590–3596. 10.4049/jimmunol.177.6.3590 16951318

[B157] Matalon O. Ben-Shmuel A. Kivelevitz J. Sabag B. Fried S. Joseph N. (2018). Actin retrograde flow controls natural killer cell response by regulating the conformation state of SHP-1. *EMBO J.* 37:e96264. 10.15252/embj.201696264 PMC5830919 29449322

[B158] Matalon O. Fried S. Ben-Shmuel A. Pauker M. H. Joseph N. Keizer D. (2016). Dephosphorylation of the adaptor LAT and phospholipase C-γ by SHP-1 inhibits natural killer cell cytotoxicity. *Sci. Signal.* 9:ra54. 10.1126/scisignal.aad6182 27221712

[B159] Matalon O. Reicher B. Barda-Saad M. (2013). Wiskott-Aldrich syndrome protein–dynamic regulation of actin homeostasis: from activation through function and signal termination in T lymphocytes. *Immunol. Rev.* 256 10–29. 10.1111/imr.12112 24117810

[B160] McCann F. E. Vanherberghen B. Eleme K. Carlin L. M. Newsam R. J. Goulding D. (2003). The size of the synaptic cleft and distinct distributions of filamentous actin, ezrin, CD43, and CD45 at activating and inhibitory human NK cell immune synapses. *J. Immunol.* 170 2862–2870. 10.4049/jimmunol.170.6.2862 12626536

[B161] Ménasché G. Pastural E. Feldmann J. Certain S. Ersoy F. Dupuis S. (2000). Mutations in RAB27A cause Griscelli syndrome associated with haemophagocytic syndrome. *Nat. Genet.* 25 173–176. 10.1038/76024 10835631

[B162] Mentlik A. N. Sanborn K. B. Holzbaur E. L. Orange J. S. (2010). Rapid lytic granule convergence to the MTOC in natural killer cells is dependent on dynein but not cytolytic commitment. *Mol. Biol. Cell* 21 2241–2256. 10.1091/mbc.e09-11-0930 20444980 PMC2893988

[B163] Miki H. Suetsugu S. Takenawa T. (1998). WAVE, a novel WASP-family protein involved in actin reorganization induced by Rac. *EMBO J.* 17 6932–6941. 10.1093/emboj/17.23.6932 9843499 PMC1171041

[B164] Mizesko M. C. Banerjee P. P. Monaco-Shawver L. Mace E. M. Bernal W. E. Sawalle-Belohradsky J. (2013). Defective actin accumulation impairs human natural killer cell function in patients with dedicator of cytokinesis 8 deficiency. *J. Allergy Clin. Immunol.* 131 840–848. 10.1016/j.jaci.2012.12.1568 23380217 PMC3646579

[B165] Monks C. R. Freiberg B. A. Kupfer H. Sciaky N. Kupfer A. (1998). Three-dimensional segregation of supramolecular activation clusters in T cells. *Nature* 395 82–86. 10.1038/25764 9738502

[B166] Moretta L. Bottino C. Pende D. Mingari M. C. Biassoni R. Moretta A. (2002). Human natural killer cells: their origin, receptors and function. *Eur. J. Immunol.* 32:1205 10.1002/1521-4141(200205)32:5<1205::aid-immu1205>3.0.co;2-y 11981807

[B167] Morin N. A. Oakes P. W. Hyun Y.-M. Lee D. Chin Y. E. King M. R. (2008). Nonmuscle myosin heavy chain IIA mediates integrin LFA-1 de-adhesion during T lymphocyte migration. *J. Exp. Med.* 205 195–205. 10.1084/jem.20071543 18195072 PMC2234359

[B168] Mukherjee S. Kim J. Mooren O. L. Shahan S. T. Cohan M. Cooper J. A. (2015). Role of cortactin homolog HS1 in transendothelial migration of natural killer cells. *PLoS One* 10:e0118153. 10.1371/journal.pone.0118153 25723543 PMC4344232

[B169] Mullins R. D. Heuser J. A. Pollard T. D. (1998). The interaction of Arp2/3 complex with actin: nucleation, high affinity pointed end capping, and formation of branching networks of filaments. *Proc. Natl. Acad. Sci. U.S.A.* 95 6181–6186. 10.1073/pnas.95.11.6181 9600938 PMC27619

[B170] Murugesan S. Hong J. Yi J. Li D. Beach J. R. Shao L. (2016). Formin-generated actomyosin arcs propel T cell receptor microcluster movement at the immune synapse. *J. Cell Biol.* 215 383–399. 10.1083/jcb.201603080 27799367 PMC5100289

[B171] Nagle D. L. Karim M. A. Woolf E. A. Holmgren L. Bork P. Misumi D. J. (1996). Identification and mutation analysis of the complete gene for chediak-higashi syndrome. *Nat. Genet.* 14 307–311. 10.1038/ng1196-307 8896560

[B172] Nakajima H. Cella M. Langen H. Friedlein A. Colonna M. (1999). Activating interactions in human NK cell recognition: the role of 2B4-CD48. *Eur. J. Immunol.* 29 1676–1683. 10.1002/(sici)1521-4141(199905)29:05<1676::aid-immu1676>3.0.co;2-y 10359122

[B173] Nolz J. C. Gomez T. S. Zhu P. Li S. Medeiros R. B. Shimizu Y. (2006). The WAVE2 complex regulates actin cytoskeletal reorganization and CRAC-mediated calcium entry during T cell activation. *Curr. Biol.* 16 24–34. 10.1016/j.cub.2005.11.036 16401421 PMC1779663

[B174] Nolz J. C. Medeiros R. B. Mitchell J. S. Zhu P. Freedman B. D. Shimizu Y. (2007). WAVE2 regulates high-affinity integrin binding by recruiting vinculin and talin to the immunological synapse. *Mol. Cell. Biol.* 27 5986–6000. 10.1128/mcb.00136-07 17591693 PMC1952166

[B175] Nolz J. C. Nacusi L. P. Segovis C. M. Medeiros R. B. Mitchell J. S. Shimizu Y. (2008). The WAVE2 complex regulates T cell receptor signaling to integrins via Abl- and CrkL-C3G-mediated activation of Rap1. *J. Cell Biol.* 182 1231–1244. 10.1083/jcb.200801121 18809728 PMC2542481

[B176] Noy E. Fried S. Matalon O. Barda-Saad M. W. I. P. (2012). Remodeling actin behind the scenes: how WIP reshapes immune and other functions. *Int. J. Mol. Sci.* 13 7629–7647. 10.3390/ijms13067629 22837718 PMC3397550

[B177] Ogbomo H. Timm-McCann M. Barnes T. Xiang R. F. Jamil K. Ganguly A. (2018). Granule-dependent NK cell killing of cryptococcus requires kinesin to reposition the cytolytic machinery for directed cytotoxicity. *Cell Rep.* 24 3017–3032. 10.1016/j.celrep.2018.08.027 30208325

[B178] Orange J. S. (2008). Formation and function of the lytic NK-cell immunological synapse. *Nat. Rev. Immunol.* 8 713–725. 10.1038/nri2381 19172692 PMC2772177

[B179] Orange J. S. (2013). Natural killer cell deficiency. *J. Allergy Clin. Immunol.* 132 515–525. 23993353 10.1016/j.jaci.2013.07.020 PMC3917661

[B180] Orange J. S. Harris K. E. Andzelm M. M. Valter M. M. Geha R. S. Strominger J. L. (2003). The mature activating natural killer cell immunologic synapse is formed in distinct stages. *Proc. Natl. Acad. Sci. U.S.A.* 100 14151–14156. 10.1073/pnas.1835830100 14612578 PMC283561

[B181] Orange J. S. Ramesh N. Remold-O’Donnell E. Sasahara Y. Koopman L. Byrne M. (2002). Wiskott-Aldrich syndrome protein is required for NK cell cytotoxicity and colocalizes with actin to NK cell-activating immunologic synapses. *Proc. Natl. Acad. Sci. U.S.A.* 99 11351–11356. 10.1073/pnas.162376099 12177428 PMC123260

[B182] Orange J. S. Roy-Ghanta S. Mace E. M. Maru S. Rak G. D. Sanborn K. B. (2011). IL-2 induces a WAVE2-dependent pathway for actin reorganization that enables WASp-independent human NK cell function. *J. Clin. Invest.* 121 1535–1548. 10.1172/jci44862 21383498 PMC3069781

[B183] Orr M. T. Lanier L. L. (2010). Natural killer cell education and tolerance. *Cell* 142 847–856. 10.1016/j.cell.2010.08.031 20850008 PMC2945212

[B184] Oszmiana A. Williamson D. J. Cordoba S.-P. Morgan D. J. Kennedy P. R. Stacey K. (2016). The size of activating and inhibitory killer Ig-like receptor nanoclusters is controlled by the transmembrane sequence and affects signaling. *Cell Rep.* 15 1957–1972. 10.1016/j.celrep.2016.04.075 27210755 PMC4893158

[B185] Pageon S. V. Aquino G. Lagrue K. Köhler K. Endres R. G. Davis D. M. (2013a). Dynamics of natural killer cell receptor revealed by quantitative analysis of photoswitchable protein. *Biophys. J.* 105 1987–1996. 10.1016/j.bpj.2013.09.025 24209843 PMC3824574

[B186] Pageon S. V. Cordoba S.-P. Owen D. M. Rothery S. M. Oszmiana A. Davis D. M. (2013b). Superresolution microscopy reveals nanometer-scale reorganization of inhibitory natural killer cell receptors upon activation of NKG2D. *Sci. Signal.* 6:ra62. 10.1126/scisignal.2003947 23882121

[B187] Papakonstanti E. A. Stournaras C. (2002). Association of PI-3 kinase with PAK1 leads to actin phosphorylation and cytoskeletal reorganization. *Mol. Biol. Cell* 13 2946–2962. 10.1091/mbc.02-01-0599 12181358 PMC117954

[B188] Pauker M. H. Barda-Saad M. (2011). Studies of novel interactions between Nck and VAV SH3 domains. *Commun. Integr. Biol.* 4 175–177. 10.4161/cib.4.2.14235 21655432 PMC3104571

[B189] Pauker M. H. Hassan N. Noy E. Reicher B. Barda-Saad M. (2012). Studying the dynamics of SLP-76, Nck, and Vav1 multimolecular complex formation in live human cells with triple-color FRET. *Sci. Signal.* 5:rs3. 10.1126/scisignal.2002423 22534133

[B190] Pauker M. H. Reicher B. Joseph N. Wortzel I. Jakubowicz S. Noy E. (2014). WASp family verprolin-homologous protein-2 (WAVE2) and Wiskott-Aldrich syndrome protein (WASp) engage in distinct downstream signaling interactions at the T cell antigen receptor site. *J. Biol. Chem.* 289 34503–34519. 10.1074/jbc.m114.591685 25342748 PMC4263859

[B191] Peterson M. E. Long E. O. (2008). Inhibitory receptor signaling via tyrosine phosphorylation of the adaptor Crk. *Immunity* 29 578–588. 10.1016/j.immuni.2008.07.014 18835194 PMC2639764

[B192] Ponti A. Machacek M. Gupton S. L. Waterman-Storer C. M. Danuser G. (2004). Two distinct actin networks drive the protrusion of migrating cells. *Science* 305 1782–1786. 10.1126/science.1100533 15375270

[B193] Purdy A. K. Campbell K. S. (2009). SHP-2 expression negatively regulates NK cell function. *J. Immunol.* 183 7234–7243. 10.4049/jimmunol.0900088 19915046 PMC2783243

[B194] Rak G. D. Mace E. M. Banerjee P. P. Svitkina T. Orange J. S. (2011). Natural killer cell lytic granule secretion occurs through a pervasive actin network at the immune synapse. *PLoS Biol.* 9:e1001151. 10.1371/journal.pbio.1001151 21931536 PMC3172191

[B195] Ravetch J. V. (2000). Immune inhibitory receptors. *Science* 290 84–89. 10.1126/science.290.5489.84 11021804

[B196] Reicher B. Joseph N. David A. Pauker M. H. Perl O. Barda-Saad M. (2012). Ubiquitylation-dependent negative regulation of WASp is essential for actin cytoskeleton dynamics. *Mol. Cell. Biol.* 32 3153–3163. 10.1128/mcb.00161-12 22665495 PMC3434509

[B197] Riteau B. Barber D. F. Long E. O. (2003). Vav1 phosphorylation is induced by beta2 integrin engagement on natural killer cells upstream of actin cytoskeleton and lipid raft reorganization. *J. Exp. Med.* 198 469–474. 10.1084/jem.20021995 12885870 PMC2194094

[B198] Roetynck S. Baratin M. Johansson S. Lemmers C. Vivier E. Ugolini S. (2006). Natural killer cells and malaria. *Immunol. Rev.* 214 251–263. 17100890 10.1111/j.1600-065X.2006.00446.x

[B199] Roose J. Weiss A. (2000). T cells: getting a GRP on Ras. *Nat. Immunol.* 1 275–276. 10.1038/79713 11017094

[B200] Rudnicka D. Oszmiana A. Finch D. K. Strickland I. Schofield D. J. Lowe D. C. (2013). Rituximab causes a polarization of B cells that augments its therapeutic function in NK-cell-mediated antibody-dependent cellular cytotoxicity. *Blood* 121 4694–4702. 10.1182/blood-2013-02-482570 23613524

[B201] Saitakis M. Dogniaux S. Goudot C. Bufi N. Asnacios S. Maurin M. (2017). Different TCR-induced T lymphocyte responses are potentiated by stiffness with variable sensitivity. *Elife* 6:e23190. 10.7554/eLife.23190 PMC5464771 28594327

[B202] Sakai Y. Tanaka Y. Yanagihara T. Watanabe M. Duan X. Terasawa M. (2013). The Rac activator DOCK2 regulates natural killer cell-mediated cytotoxicity in mice through the lytic synapse formation. *Blood* 122 386–393. 10.1182/blood-2012-12-475897 23719299

[B203] Salzer E. Cagdas D. Hons M. Mace E. M. Garncarz W. Petronczki ÖY. (2016). RASGRP1 deficiency causes immunodeficiency with impaired cytoskeletal dynamics. *Nat. Immunol.* 17 1352–1360. 10.1038/ni.3575 27776107 PMC6400263

[B204] Sanborn K. B. Mace E. M. Rak G. D. Difeo A. Martignetti J. A. Pecci A. (2011). Phosphorylation of the myosin IIA tailpiece regulates single myosin IIA molecule association with lytic granules to promote NK-cell cytotoxicity. *Blood* 118 5862–5871. 10.1182/blood-2011-03-344846 22123909 PMC3228501

[B205] Sanborn K. B. Rak G. D. Maru S. Y. Demers K. Difeo A. Martignetti J. A. (2009). Myosin IIA associates with NK cell lytic granules to enable their interaction with F-actin and function at the immunological synapse. *J. Immunol.* 182 6969–6984. 10.4049/jimmunol.0804337 19454694 PMC2835774

[B206] Sancho D. Nieto M. Llano M. Rodríguez-Fernández J. L. Tejedor R. Avraham S. (2000). The tyrosine kinase PYK-2/RAFTK regulates natural killer (NK) cell cytotoxic response, and is translocated and activated upon specific target cell recognition and killing. *J. Cell Biol.* 149 1249–1262. 10.1083/jcb.149.6.1249 10851022 PMC2175114

[B207] Sayos J. Wu C. Morra M. Wang N. Zhang X. Allen D. (1998). The X-linked lymphoproliferative-disease gene product SAP regulates signals induced through the co-receptor SLAM. *Nature* 395 462–469. 10.1038/26683 9774102

[B208] Schuyler S. C. Pellman D. (2001). Microtubule “Plus-End-Tracking Proteins”: the end is just the beginning. *Cell* 105 421–424. 10.1016/s0092-8674(01)00364-6 11371339

[B209] Segovis C. M. Schoon R. A. Dick C. J. Nacusi L. P. Leibson P. J. Billadeau D. D. (2009). PI3K links NKG2D signaling to a CrkL pathway involved in natural killer cell adhesion, polarity, and granule secretion. *J. Immunol.* 182 6933–6942. 10.4049/jimmunol.0803840 19454690 PMC2706535

[B210] Seri M. Pecci A. Di Bari F. Cusano R. Savino M. Panza E. (2003). MYH9-Related Disease. *Medicine* 82 203–215. 12792306 10.1097/01.md.0000076006.64510.5c

[B211] Shaheen S. Wan Z. Li Z. Chau A. Li X. Zhang S. (2017). Substrate stiffness governs the initiation of B cell activation by the concerted signaling of PKCβ and focal adhesion kinase. *eLife* 6:e23060. 10.7554/eLife.23060 PMC5536945 28755662

[B212] Sidorenko S. P. Clark E. A. (2003). The dual-function CD150 receptor subfamily: the viral attraction. *Nat. Immunol.* 4 19–24. 10.1038/ni0103-19 12496974

[B213] Sims T. N. Soos T. J. Xenias H. S. Dubin-Thaler B. Hofman J. M. Waite J. C. (2007). Opposing effects of PKCtheta and WASp on symmetry breaking and relocation of the immunological synapse. *Cell* 129 773–785. 10.1016/j.cell.2007.03.037 17512410

[B214] Sinai P. Nguyen C. Schatzle J. D. Wülfing C. (2010). Transience in polarization of cytolytic effectors is required for efficient killing and controlled by Cdc42. *Proc. Natl. Acad. Sci. U.S.A.* 107 11912–11917. 10.1073/pnas.0913422107 20547841 PMC2900700

[B215] Sproul L. R. Anderson D. J. Mackey A. T. Saunders W. S. Gilbert S. P. (2005). Cik1 targets the minus-end kinesin depolymerase kar3 to microtubule plus ends. *Curr. Biol.* 15 1420–1427. 10.1016/j.cub.2005.06.066 16085496 PMC2386176

[B216] Staaf E. Hedde P. N. Bagawath Singh S. Piguet J. Gratton E. Johansson S. (2018). Educated natural killer cells show dynamic movement of the activating receptor NKp46 and confinement of the inhibitory receptor Ly49A. *Sci. Signal.* 11:eaai9200. 10.1126/scisignal.aai9200 29440510 PMC5925740

[B217] Stabile H. Carlino C. Mazza C. Giliani S. Morrone S. Notarangelo L. D. (2010). Impaired NK-cell migration in WAS/XLT patients: role of Cdc42/WASp pathway in the control of chemokine-induced β2 integrin high-affinity state. *Blood* 115 2818–2826. 10.1182/blood-2009-07-235804 20130240 PMC2854428

[B218] Standeven L. J. Carlin L. M. Borszcz P. Davis D. M. Burshtyn D. N. (2004). The Actin cytoskeleton controls the efficiency of killer Ig-Like receptor accumulation at inhibitory NK Cell immune synapses. *J. Immunol.* 173 5617–5625. 10.4049/jimmunol.173.9.5617 15494512

[B219] Stanton R. J. Prod’homme V. Purbhoo M. A. Moore M. Aicheler R. J. Heinzmann M. (2014). HCMV pUL135 remodels the actin cytoskeleton to impair immune recognition of infected cells. *Cell Host Microbe* 16 201–214. 10.1016/j.chom.2014.07.005 25121749 PMC4150922

[B220] Stebbins C. C. Watzl C. Billadeau D. D. Leibson P. J. Burshtyn D. N. Long E. O. (2003). Vav1 dephosphorylation by the tyrosine phosphatase SHP-1 as a mechanism for inhibition of cellular cytotoxicity. *Mol. Cell. Biol.* 23 6291–6299. 10.1128/mcb.23.17.6291-6299.2003 12917349 PMC180957

[B221] Stepp S. E. Dufourcq-Lagelouse R. Le Deist F. Bhawan S. Certain S. Mathew P. A. (1999). Perforin gene defects in familial hemophagocytic lymphohistiocytosis. *Science* 286 1957–1959. 10.1126/science.286.5446.1957 10583959

[B222] Stinchcombe J. C. Majorovits E. Bossi G. Fuller S. Griffiths G. M. (2006). Centrosome polarization delivers secretory granules to the immunological synapse. *Nature* 443 462–465. 10.1038/nature05071 17006514

[B223] Stone J. C. Dower N. A. Stang S. L. Bottorff D. A. Ebinu J. O. Dickie P. (2000). RasGRP is essential for mouse thymocyte differentiation and TCR signaling. *Nat. Immunol.* 1 317–321. 10.1038/79766 11017103

[B224] Suetsugu S. Miki H. Takenawa T. (1999). Identification of two human WAVE/SCAR homologues as general actin regulatory molecules which associate with the Arp2/3 complex. *Biochem. Biophys. Res. Commun.* 260 296–302. 10.1006/bbrc.1999.0894 10381382

[B225] Sullivan K. E. Mullen C. A. Blaese R. M. Winkelstein J. A. (1994). A multiinstitutional survey of the Wiskott-Aldrich syndrome. *J. Pediatr.* 125 876–885. 10.1016/s0022-3476(05)82002-5 7996359

[B226] Takeda K. (1993). The development of autoimmunity in C57BL/6 lpr mice correlates with the disappearance of natural killer type 1-positive cells: evidence for their suppressive action on bone marrow stem cell proliferation, B cell immunoglobulin secretion, and autoimmune sy. *J. Exp. Med.* 177 155–164. 10.1084/jem.177.1.155 8418197 PMC2190856

[B227] Takenawa T. Suetsugu S. (2007). The WASP-WAVE protein network: connecting the membrane to the cytoskeleton. *Nat. Rev. Mol. Cell Biol.* 8 37–48. 10.1038/nrm2069 17183359

[B228] Tapon N. (1997). Rho, Rac and Cdc42 GTPases regulate the organization of the Actin cytoskeleton. *Curr. Opin. Cell Biol.* 9 86–92. 10.1016/s0955-0674(97)80156-1 9013670

[B229] Thomas L. M. Peterson M. E. Long E. O. (2013). Cutting edge: NK cell licensing modulates adhesion to target cells. *J. Immunol.* 191 3981–3985. 10.4049/jimmunol.1301159 24038086 PMC3795972

[B230] Thrasher A. J. Burns S. O. (2010). WASP: a key immunological multitasker. *Nat. Rev. Immunol.* 10 182–192. 10.1038/nri2724 20182458

[B231] Timonen T. (1997). Natural killer cells: endothelial interactions, migration, and target cell recognition. *J. Leukoc. Biol.* 62 693–701. 10.1002/jlb.62.6.693 9400809

[B232] Tojkander S. Gateva G. Lappalainen P. (2012). Actin stress fibers - Assembly, dynamics and biological roles. *J. Cell Sci.* 125 1855–1864. 10.1242/jcs.098087 22544950

[B233] Topham N. J. Hewitt E. W. (2009). Natural killer cell cytotoxicity: how do they pull the trigger? *Immunology* 128 7–15. 10.1111/j.1365-2567.2009.03123.x 19689731 PMC2747134

[B234] Treanor B. Lanigan P. M. P. Kumar S. Dunsby C. Munro I. Auksorius E. (2006). Microclusters of inhibitory killer immunoglobulin-like receptor signaling at natural killer cell immunological synapses. *J. Cell Biol.* 174 153–161. 10.1083/jcb.200601108 16801390 PMC2064172

[B235] Tsukita S. Yonemura S. (1999). Cortical actin organization: lessons from ERM (ezrin/radixin/moesin) proteins. *J. Biol. Chem.* 274 34507–34510. 10.1074/jbc.274.49.34507 10574907

[B236] Tuli A. Thiery J. James A. M. Michelet X. Sharma M. Garg S. (2013). Arf-like GTPase Arl8b regulates lytic granule polarization and natural killer cell-mediated cytotoxicity. *Mol. Biol. Cell* 24 3721–3735. 10.1091/mbc.e13-05-0259 24088571 PMC3842998

[B237] Upshaw J. L. Arneson L. N. Schoon R. A. Dick C. J. Billadeau D. D. Leibson P. J. (2006). NKG2D-mediated signaling requires a DAP10-bound Grb2-Vav1 intermediate and phosphatidylinositol-3-kinase in human natural killer cells. *Nat. Immunol.* 7 524–532. 10.1038/ni1325 16582911

[B238] Varma R. Campi G. Yokosuka T. Saito T. Dustin M. L. (2006). T cell receptor-proximal signals are sustained in peripheral microclusters and terminated in the central supramolecular activation cluster. *Immunity* 25 117–127. 10.1016/j.immuni.2006.04.010 16860761 PMC1626533

[B239] Vély F. Vivier E. (2005). Natural killer cell receptor signaling pathway. *Sci. Signal.* 2005:cm6. 10.1126/stke.2922005cm6 16014603

[B240] Viant C. Fenis A. Chicanne G. Payrastre B. Ugolini S. Vivier E. (2014). SHP-1-mediated inhibitory signals promote responsiveness and anti-tumour functions of natural killer cells. *Nat. Commun.* 5:5108. 10.1038/ncomms6108 25355530

[B241] Vicente-Manzanares M. Ma X. Adelstein R. S. Horwitz A. R. (2009). Non-muscle myosin II takes centre stage in cell adhesion and migration. *Nat. Rev. Mol. Cell Biol.* 10 778–790. 10.1038/nrm2786 19851336 PMC2834236

[B242] Vicente-Manzanares M. Sánchez-Madrid F. (2004). Role of the cytoskeleton during leukocyte responses. *Nat. Rev. Immunol.* 4 110–122. 10.1038/nri1268 15040584

[B243] Villegas F. R. Coca S. Villarrubia V. G. Jiménez R. Chillón M. J. Jareño J. (2002). Prognostic significance of tumor infiltrating natural killer cells subset CD57 in patients with squamous cell lung cancer. *Lung Cancer* 35 23–28. 10.1016/s0169-5002(01)00292-6 11750709

[B244] Vivier E. Raulet D. H. Moretta A. Caligiuri M. A. Zitvogel L. Lanier L. L. (2011). Innate or adaptive immunity? The example of natural killer cells. *Science* 331 44–49. 21212348 10.1126/science.1198687 PMC3089969

[B245] Vivier E. Tomasello E. Baratin M. Walzer T. Ugolini S. (2008). Functions of natural killer cells. *Nat. Immunol.* 9 503–510. 18425107 10.1038/ni1582

[B246] Vyas Y. M. Maniar H. Dupont B. (2002a). Cutting edge: differential segregation of the SRC homology 2-containing protein tyrosine phosphatase-1 within the early NK cell immune synapse distinguishes noncytolytic from cytolytic interactions. *J. Immunol.* 168 3150–3154. 10.4049/jimmunol.168.7.3150 11907066

[B247] Vyas Y. M. Maniar H. Dupont B. (2002b). Visualization of signaling pathways and cortical cytoskeleton in cytolytic and noncytolytic natural killer cell immune synapses. *Immunol. Rev.* 189 161–178. 10.1034/j.1600-065x.2002.18914.x 12445273

[B248] Vyas Y. M. Mehta K. M. Morgan M. Maniar H. Butros L. Jung S. (2001). Spatial organization of signal transduction molecules in the NK cell immune synapses during MHC class I-regulated noncytolytic and cytolytic interactions. *J. Immunol.* 167 4358–4367. 10.4049/jimmunol.167.8.4358 11591760

[B249] Wagtmann N. Rajagopalan S. Winter C. C. Peruui M. Long E. O. (1995). Killer cell inhibitory receptors specific for HLA-C and HLA-B identified by direct binding and by functional transfer. *Immunity* 3 801–809. 10.1016/1074-7613(95)90069-1 8777725

[B250] Wallar B. J. Alberts A. S. (2003). The formins: active scaffolds that remodel the cytoskeleton. *Trends Cell Biol.* 13 435–446. 10.1016/s0962-8924(03)00153-3 12888296

[B251] Walzer T. Dalod M. Robbins S. H. Zitvogel L. Vivier E. (2005). Natural-killer cells and dendritic cells: ‘l’union fait la force’. *Blood* 106 2252–2258. 10.1182/blood-2005-03-1154 15933055

[B252] Wang Y. Sun J. Ma C. Gao W. Song B. Xue H. (2016). Reduced expression of Galectin-9 contributes to a poor outcome in colon cancer by inhibiting NK cell chemotaxis partially through the Rho/ROCK1 signaling pathway. *PLoS One* 11:e0152599. 10.1371/journal.pone.0152599 27028892 PMC4814049

[B253] Watzl C. Long E. O. (2003). Natural killer cell inhibitory receptors block actin cytoskeleton-dependent recruitment of 2B4 (CD244) to lipid rafts. *J. Exp. Med.* 197 77–85. 10.1084/jem.20020427 12515815 PMC2193803

[B254] Watzl C. Long E. O. (2010). Signal transduction during activation and inhibition of natural killer cells. *Curr. Protoc. Immunol.* 11:Unit11.9B. 10.1002/0471142735.im1109bs90 PMC3857016 20814939

[B255] Watzl C. Stebbins C. C. Long E. O. (2000). Cutting edge: NK cell inhibitory receptors prevent tyrosine phosphorylation of the activation receptor 2B4 (CD244). *J. Immunol.* 165 3545–3548. 10.4049/jimmunol.165.7.3545 11034353

[B256] Wilton K. M. Billadeau D. D. V. A. S. P. (2018). Regulates NK cell lytic granule convergence. *J. Immunol.* 201 2899–2909. 10.4049/jimmunol.1800254 30282752 PMC6317751

[B257] Wilton K. M. Overlee B. L. Billadeau D. D. (2019). NKG2D-DAP10 signaling recruits EVL to the cytotoxic synapse to generate F-actin and promote NK cell cytotoxicity. *J. Cell Sci.* 133:jcs230508. 10.1242/jcs.230508 31235500 PMC7055393

[B258] Wood S. M. Meeths M. Chiang S. C. C. Bechensteen A. G. Boelens J. J. Heilmann C. (2009). Different NK cell-activating receptors preferentially recruit Rab27a or Munc13-4 to perforin-containing granules for cytotoxicity. *Blood* 114 4117–4127. 10.1182/blood-2009-06-225359 19704116

[B259] Worthylake R. A. Burridge K. (2001). Leukocyte transendothelial migration: Orchestrating the underlying molecular machinery. *Curr. Opin. Cell Biol.* 13 569–577. 10.1016/s0955-0674(00)00253-2 11544025

[B260] Wulfing C. Purtic B. Klem J. Schatzle J. D. (2003). Stepwise cytoskeletal polarization as a series of checkpoints in innate but not adaptive cytolytic killing. *Proc. Natl. Acad. Sci. U.S.A.* 100 7767–7772. 10.1073/pnas.1336920100 12802007 PMC164662

[B261] Yi J. Wu X. S. Crites T. Hammer J. A. (2012). Actin retrograde flow and actomyosin II arc contraction drive receptor cluster dynamics at the immunological synapse in Jurkat T cells. *Mol. Biol. Cell* 23 834–852. 10.1091/mbc.e11-08-0731 22219382 PMC3290643

[B262] Zamai L. Ahmad M. Bennett I. M. Azzoni L. Alnemri E. S. Perussia B. (1998). Natural Killer (NK) cell-mediated cytotoxicity: differential use of ?TRAIL and Fas ligand by immature and mature primary human NK cells. *J. Exp. Med.* 188:2375. 10.1084/jem.188.12.2375 9858524 PMC2212426

[B263] Zanic M. Widlund P. O. Hyman A. A. Howard J. (2013). Synergy between XMAP215 and EB1 increases microtubule growth rates to physiological levels. *Nat. Cell Biol.* 15 688–693. 10.1038/ncb2744 23666085

[B264] Zhang M. March M. E. Lane W. S. Long E. O. (2014). A signaling network stimulated by 2 integrin promotes the polarization of lytic granules in cytotoxic cells. *Sci. Signal.* 7:ra96. 10.1126/scisignal.2005629 25292215 PMC4205566

[B265] Zhang Q. Davis J. C. Lamborn I. T. Freeman A. F. Jing H. Favreau A. J. (2009). Combined immunodeficiency associated with DOCK8 mutations. *N. Engl. J. Med.* 361 2046–2055. 19776401 10.1056/NEJMoa0905506 PMC2965730

[B266] Zhang Z. Wu N. Lu Y. Davidson D. Colonna M. Veillette A. (2015). DNAM-1 controls NK cell activation via an ITT-like motif. *J. Exp. Med.* 212 2165–2182. 10.1084/jem.20150792 26552706 PMC4647266

